# Community-based eDNA metabarcoding for monitoring fish biodiversity and food webs in the Peace-Athabasca Delta

**DOI:** 10.7717/peerj.21341

**Published:** 2026-05-22

**Authors:** Yoamel Milián-García, Paul Giroux, Margaret Docker, Arfa Khan, Ken M. Jeffries, Kate Lindsay, Queenie Gray, Morgan Voyageur, Bruce Maclean, Caroline Bampfylde, Cassandre Pyne, Robert H. Hanner

**Affiliations:** 1 Department of Integrative Biology, University of Guelph, Guelph, Ontario, Canada; 2 Ecological and Regulatory (Ecoreg) Solutions Inc., Guelph, Ontario, Canada; 3 Wood Buffalo National Park of Canada World Heritage Site Action Plan, Office of the Chief Ecosystem Scientist, Protected Areas, Establishment and Conservation, Parks Canada, Gatineau, Quebec, Canada; 4 Department of Biological Sciences, University of Manitoba, Winnipeg, Manitoba, Canada; 5 Fisheries and Ocean Canada, Winnipeg, Manitoba, Canada; 6 Parks Canada, Fort Smith, Northwest Territories, Canada; 7 Athabasca Chipewyan First Nation, Dené Lands and Resource Management, Fort Chipewyan, Canada; 8 Nipîy Tu Research and Knowledge Centre, Fort Chipewyan, Canada; 9 Department of Ecology and Evolutionary Biology, University of Toronto, Toronto, Ontario, Canada

**Keywords:** eDNA metabarcoding, Fish, 12S, COI, Peace-Athabasca Delta (PAD), Wood Buffalo National Park (WBNP), Food Web, First Nation, Fort Chipewyan

## Abstract

The Peace-Athabasca Delta (PAD) is the largest freshwater inland delta in North America. It holds Outstanding Universal Value (OUV) as part of the Wood Buffalo National Park (WBNP) World Heritage Site (UNESCO). The health of the PAD and its OUV was questioned by the Mikisew Cree First Nation in a petition to UNESCO in 2014. In response, UNESCO directed Canada to take a series of actions to determine if WBNP should be listed as a World Heritage in danger. Canada created the WBNP Action Plan (2018), which included a key action to assess the condition of WBNP’s OUV with local First Nations. The waters of the delta have been and remain the habitat of essential fishery resources for the Indigenous Communities in the area. There is, however, limited scientific data available concerning the abundance and distribution of fish, their health, and the structure of the food webs that support them within the PAD. Thus, innovative molecular techniques, such as environmental DNA (eDNA) metabarcoding, can enhance existing capture-based sampling methods for assessing and monitoring freshwater ecosystems. A pilot project was conducted in the summer and fall of 2021 to evaluate the potential utility of molecular tools in addressing key knowledge gaps regarding fish diversity, distribution, and contributing to the collaborative Integrated Research and Monitoring Program’s assessment of PAD health. In this context, eDNA metabarcoding methods were employed to assess community diversity from traces of DNA that organisms shed into the aquatic environment or release during digestion (prey DNA). Samples were collected in collaboration with local Indigenous Governments, specifically the Athabasca Chipewyan and Mikisew Cree First Nations’ Community-Based Monitoring (CBM) technical staff and the Mikisew Cree Land-Users Advocacy Network (LUAN), Parks Canada, and the Genomic Network for Fish Identification, Stress and Health (GEN-FISH). Using two mitochondrial markers (COI and 12S), eDNA metabarcoding studies detected a combined total of 15 fish species known to inhabit PAD waters, with distinct distributions across the 15 sampling locations. Differential performance in fish detection was observed between the molecular markers used, with 12S providing greater specificity for fish and COI providing broader taxonomic coverage, detecting a diversity of birds, mammals, and invertebrates from the region. Similarly, DNA metabarcoding analysis of stomach contents of Lake Whitefish (*Coregonus clupeaformis*) detected nine of 19 previously reported diet items, including all the primary known diet items for Lake Whitefish, except Sphaeriids and *Pontoporeia.* The above results highlight the utility and readiness level of the molecular tools developed by GEN-FISH for conducting fish surveys and assessing food webs in freshwater at sites surrounding Fort Chipewyan.

## Introduction

The Peace-Athabasca Delta (PAD) is considered one of the largest freshwater inland deltas in the world and the largest in North America, occupying approximately 6,000 km^2^ (Peters et al., 2006). This complex and dynamic ecosystem serves as the habitat for multiple species and the homeland of the region’s Indigenous Communities (Parks Canada Agency, 2018). The PAD is also a Ramsar wetland, protected within the Wood Buffalo National Park (WBNP). WBNP of Canada was inscribed as a UNESCO World Heritage site in 1983, and the Peace-Athabasca Delta was listed as one of the park’s outstanding universal values (OUV) (IUCN, 1983). The PAD is largely contained within WBNP of Canada. A portion of the Athabasca River delta, south of the Embarras River, is outside of the Park and is administered by the Government of Alberta and local First Nations. In 2014, the Mikisew Cree First Nation submitted a petition to UNESCO to have WBNP of Canada listed as a UNESCO World Heritage Site in danger due to substantial hydrologic impacts to the PAD associated with the WAC Bennett Dam on the Peace River and pollution from Oil Sands Mining within the Athabasca River watershed (Cree, 2014). Following a 2016 UNESCO Reactive Monitoring Mission, Canada was instructed, among other items, to work collaboratively with local Indigenous Governments and to apply Indigenous Knowledge (IK) in the implementation of research and monitoring activities involving assessments of PAD health. A pilot study applying emerging eDNA methods represents an effort to use innovative techniques in conjunction with IK to assess local fish population distribution and health as part of the Wood Buffalo National Park Action Plan’s Integrated Research and Monitoring Program for the PAD.

The abundant natural resources of the PAD have provided sustenance, a way of life, and culture for the local Indigenous Communities for many generations (Parks Canada Agency, 2022). Fisheries are among the traditional activities conducted by the Indigenous Communities (Parks Canada Agency, 2022), in addition to (now closed) commercial fishing since 1954 (Timoney, 2009). Walleye (*Sander vitreus*), Lake Whitefish (*Coregonus clupeaformis*), Goldeye (*Hiodon alosoides*) and Northern Pike (*Esox lucius*) are among the fish with the highest importance to local fishers (Timoney, 2009). At Fort Chipewyan, fish is a staple of the local food supply, involving most inhabitants (Timoney, 2009). Present-day fishers collect fish for human consumption and to feed dog teams, with seasonal collection and preparation of dried Lake Whitefish maintained as a traditional community practice. The time spent by the community in this cultural practice is utilized by the PAD Nations’ Community-Based Monitoring (CBM) programs to gather scientific and IK about fish behaviour, movements, and health. Despite these efforts, there remains limited Western scientific knowledge of fish populations targeted by fishers, including fish diversity, feeding behaviour, seasonal movements, abundance, and health, which hampers food security (Timoney, 2009).

Modern molecular techniques, such as environmental DNA (eDNA) metabarcoding, offer valuable biomonitoring tools, allowing for the detection of species in water bodies through non-invasive sampling and minimal ecological disturbance. Similarly, this molecular-based identification method can be applied to food web characterization for a fish species of interest, cultural importance, or concern, and can provide additional information that might be linked to feeding behaviour. Consequently, this molecular technique is revolutionizing biodiversity monitoring while overcoming widespread limitations (*e.g.*, time-consuming, scarcity of expert taxonomists, need for morphological integrity) of traditional methods (*e.g.*, morphology-based identifications). The straightforward nature of eDNA sample collection requires minimal training and can be readily transferred to existing community-based monitoring projects.

Consequently, short DNA sequences (*e.g.*, DNA barcodes) can be used for multi-taxa identification when paired with High-Throughput eDNA Sequencing (eDNA metabarcoding), which has revolutionized biodiversity monitoring, offering a powerful molecular tool for evaluating species richness in almost any environment (Deiner et al., 2017). As a DNA barcoding-based molecular technique, it uses established DNA reference sequence databases to conduct multi-taxa identification, enabling rapid and cost-effective molecular-based taxonomic assignment of unknown to known sequences (Milián-García et al., 2020; Milián-García et al., 2025). This molecular technique can base species identifications on DNA extracted from bulk tissue samples or by non-invasively sampling DNA shed by the organisms as they interact with their environment (eDNA). Thus, eDNA metabarcoding can complement and surpass traditional species identification methods by enabling the detection of multiple species, identifying target species in the context of non-target species, and providing higher taxonomic resolution than conventional methods (Deiner et al., 2017; Carvalho et al., 2021). Consequently, eDNA metabarcoding has proven highly effective in fish identification (Miya et al., 2015; Sato et al., 2018) and in revealing the structure of food webs (Mychek-Londer, Chaganti & Heath, 2020). Therefore, two main objectives were proposed as part of a pilot study in waters in and around the Peace-Athabasca Delta: (a) to determine what fish species are present at sites surrounding Fort Chipewyan at a particular point in time; (b) to identify the species in the stomachs of Lake Whitefish captured at the 2021 Community-Based Monitoring (CBM) Fish Camp hosted at Dog Camp (WBNP).

## Materials & Methods

### Fish community & biodiversity monitoring (water sample collection)

The GEN-FISH eDNA sampling protocol utilizes a cordless drill to drive a peristaltic pump (https://gen-fish.ca/wp-content/uploads/2022/06/DIY-Peristaltic-Pump_Protocol-and-Checklist_30JUNE2022.pdf), which draws a water sample through a filter (no standardized flow rate) to capture eDNA for further analysis. This method was based on a protocol previously described in the literature (Goldberg et al., 2011). It is suitable for use in the field in partnership with local community-based monitoring programs because it is affordable and compact, and only modest training is required to collect eDNA samples using this method. This provides an opportunity to assess the usefulness of the tools, gain feedback, increase reception, and refine the ease of use for future community-based eDNA monitoring. Notably, eDNA sampling can be coupled with conventional capture-based fish monitoring methods to provide either visual confirmation that species captured are reflected in eDNA sampling or to reveal false negatives due to taxonomic blind spots (Morey et al., 2024). These findings may indicate the presence of species that could evade detection using conventional methods or highlight the limitations of molecular tools in identifying specific species known to be present in the environment. Initial demonstrations and training sessions were conducted at the Athabasca Chipewyan First Nation (ACFN) Youth-Elder Lodge on Wednesday, August 18, 2021, to ensure that all field sample collection teams were familiar with clean sampling techniques at and between each sampling site. To facilitate hands-on training of multiple components, we divided into smaller groups. This allowed everyone to practice filtering with tap water, share their experiences and knowledge, and better cater to the locations where we sampled for eDNA, while also getting to know each other. Groups were shown techniques of how to collect eDNA water samples using bottles to prevent and minimize cross-contamination, how to filter eDNA samples using a cordless drill operated “do it yourself” (DIY) peristaltic pump system, decontaminating sampling equipment, and how to store the collected filter sample in a self-indicating silica securely. In addition, sample metadata recording instructions and sheets for both lentic and lotic water bodies were provided to ensure that sample information could be traced directly back to the collection site. All shared protocols and sheets were developed by GEN-FISH. Printouts of sampling protocols and metadata sheets were shared with the smaller groups to illustrate the step-by-step process of sampling that could be taken into the field and used as a reference for future sampling. The shared eDNA sampling protocols and metadata sheets have been included in Appendix S1.

After the hands-on demonstrations, three smaller groups travelled by boat to the north, west, and southeast of Fort Chipewyan. All the sites selected for eDNA sampling were chosen with the input and knowledge shared by Fort Chipewyan community members, Parks Canada staff in the field, and ACFN CBM staff. Historical capture information from previous fishing and fish camps for species of particular interest to the community helped determine ideal locations for eDNA collection. Water samples were collected in the field, kept in a cooler, and filtered immediately upon return to the ACFN Youth-Elder Lodge, as filtering on boats proved unstable due to strong winds. Additional water bodies and areas of interest identified by the community, ACFN CBM, and Parks Canada were sampled on Thursday, August 19, 2021, to ensure a comprehensive coverage of various areas. Big Egg Lake was identified as an area of interest but was not sampled on August 19, as access by boat, as indicated by Morgan Voyageur, is usually challenging. Still, there were additional weather and time limitations. In this regard, supplies and consumables were provided to ACFN CBM, enabling opportunistic sampling of eDNA at Big Egg Lake, which was completed during an independent sampling event on September 12, 2021.

**Figure 1:**
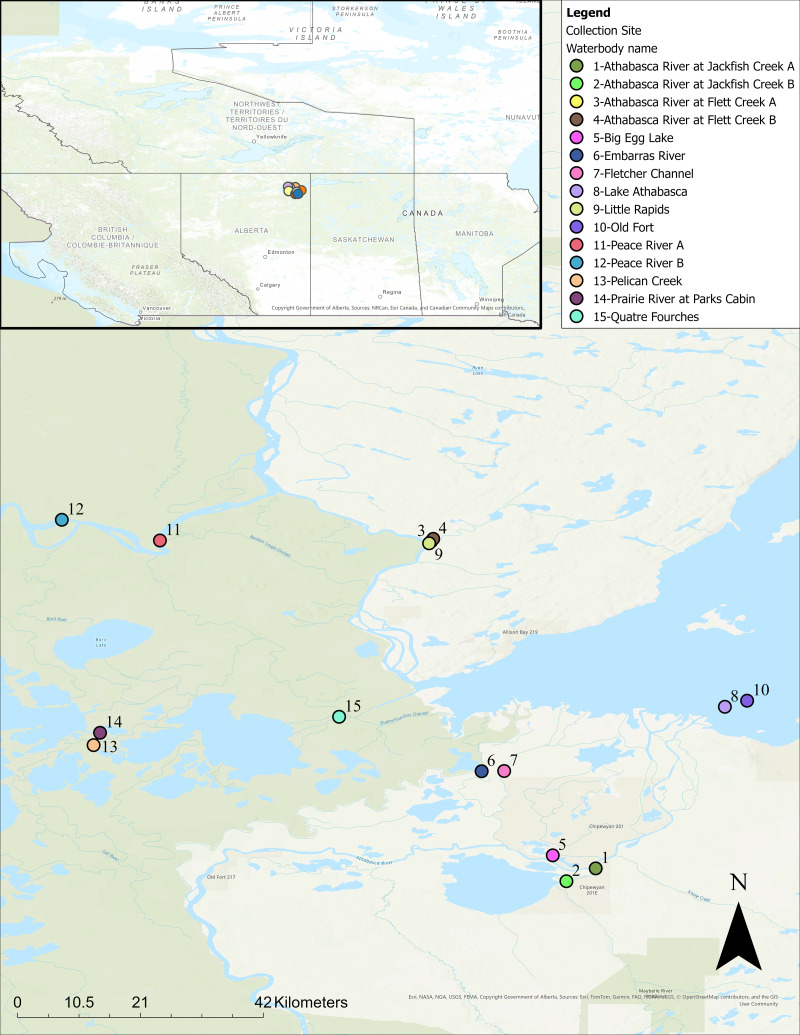
Map showing the distribution of the 15 collection sites for the water samples. An interactive version of the map is also provided in the Supplemental Information.

The Hanner Lab at the University of Guelph received three replicate filters (*i.e.,* biological replicates) and one negative control from 15 different eDNA sampling locations (Fig. 1), along with two tap water samples, totalling 62 samples. All samples were collected under Parks Canada permit No.: WB-2021-39880. Each filter was stored in a separate coin envelope and Ziploc bags with self-indicating silica beads (one for each sampling site). In addition, the Hanner Laboratory received 14 fin clips from fish captured in the local domestic fishery at Jackfish Creek, Peace River, and Quatre Fourches for individual DNA barcoding and molecular testing. All the fish sampled were morphologically identified by the fisher at the time of sampling.

### eDNA extraction and metabarcoding library preparation

eDNA was extracted from one filter (split into halves) from each collection site, including the field negative control, using a modified protocol for the Qiagen DNeasy Blood and Tissue kit (Milián-García, 2022). Each filter was allowed to thaw and cut into quarters using a sterile razor blade. Filter quarters were then cut into small pieces, which were placed in individual 2 ml microcentrifuge tubes with approximately 250 mg of glass beads (0.75–1 mm diameter). Then, 380 µL of Buffer ATL was added to each tube, and the tubes were disrupted using the Qiagen TissueLyser for 1 min at 30 Hz.

Samples were spun down, and 20 μL of Proteinase K was added to each microcentrifuge tube. After vortexing, the tubes were incubated at 56 °C overnight. The tubes were then vortexed for 15 s and spun down. Then, 400 μL of Buffer AL was added to each tube. Samples were then vortexed for 10 s and incubated at 56 °C for another 10 min. The entire lysate, including the remaining filter, was homogenized using the QIAShredder (Qiagen) by spinning it through the columns at 20,000 g for 2 min. After separating the lysate from the remaining filter, 400 μL of ethanol (96–100%) was added to each tube, and these were vortexed again for another 30 s. All samples, including any residues, were then pipetted into the DNeasy Mini spin columns and placed into 2 ml collection tubes. These were then centrifuged at 11,000 g for one minute. The columns were transferred into new collection tubes and washed twice according to the manufacturer’s protocol, using AW1 and AW2 buffers (500 μL each) for the first and second washes, respectively. Samples were centrifuged at 11,000 g for one minute after the first wash and then centrifuged at 17,000 g for 5 min after the second wash. Flow-through and collection tubes were discarded. The DNeasy Mini spin columns were then placed into clean 1.5 ml microcentrifuge tubes (LoBind Eppendorf). A total of 200 μL of Buffer AE (pre-warmed at 70 °C) was added directly onto the DNeasy spin column membranes, incubated at room temperature for 5 min, and centrifuged for final elution for one minute at 11,000 g.

eDNA extracts from each quarter belonging to the same trap were pooled into a single tube. After eDNA concentrations were determined, a five μL subsample was used to visually assess the presence and quality of eDNA on a 1% agarose gel electrophoresis. All eDNA extracts were stored at 4 °C for immediate use or at −20 °C for short-term storage prior to library preparation for High-Throughput Sequencing.

All DNA extractions were quantified three times (every 10 min) using the HS Qubit kit (Thermo Fisher), and the average concentration and standard deviation were calculated to determine the success of the DNA extractions accurately.

#### Fish DNA barcoding

For standard COI barcoding of stomach tissues and fin clips, we used the forward cocktail, comprising VF2_t1 and FishF2_t1 primers, and the reverse cocktail, comprising FR1d_t1 and FishR2_t1 (Ward et al., 2005; Ivanova et al., 2007). Each reaction contained 12.5 μL of 2X KAPA HiFi HotStart ReadyMix (Roche Diagnostics), five μL of each primer (one μM), and 2.5 μL of the DNA extract. Primer sequences and polymerase chain reaction (PCR) programs matched those reported in the literature (Ivanova et al., 2007). The primer components are labelled “component name_t”, where _t indicates they’ve been modified to include an M13 sequencing tag. Bidirectional Sanger sequencing was performed using the M13 tails. The raw sequence data were verified, low-quality ends trimmed, and the forward and reverse sequences assembled into a contiguous sequence using Geneious Prime 2026.0.2 (https://www.geneious.com/).

**Table 1:** List of primers used for each method, including primer sequences, PCR conditions, and citation. Underscored sequences represent M13 tags and Illumina adapters as used for DNA barcoding and metabarcoding primers, respectively.

**Primer**	**Method**	**Sequence 5′-3′**	**Marker**	**PCR conditions**	**Citation**
MiFish-Ur-F	eDNA Metabarcoding	TCGTCGGCAGCGTCAGATGTGT ATA AGAGACAGCCGGTA AAACTCGTGCCAGC	12S	95 ^∘^C (180 s), followed by 35 cycles of 98 ^∘^C (20 s), 65 ^∘^C (15 s), 72 ^∘^C (15 s), and a final extension at 72 ^∘^C (5 min)	Miya, Gotoh & Sado, 2020
MiFish-U-R	eDNA Metabarcoding	GTCTCGTGGGCTCGGAGATGTG TATAAGAGACAGCATAGTGGGGTA TCTAATCCCAGTTTG	12S		
VertCOI-F1_t1	eDNA Metabarcoding	TCGTCGGCAGCGTCAGATGTGT ATAAGAGACAGACCACWATTATT AAY ATAAARCCMC	COI	95 ^∘^C (120 s) followed by 60 cycles at 95 ^∘^C (40 s), 56 ^∘^C (40 s), 72 ^∘^C (30 s); and a final extension at 72 ^∘^C (5 min)	González et al., 2020
VertCOI-F2_t1	eDNA Metabarcoding	TCGTCGGCAGCGTCAGATGTGT ATAAGAGACAGACTACAGCAATT AACATAAAACCMC	COI		
VertCOI-VR1_t1	eDNA Metabarcoding	GTCTCGTGGGCTCGGAGATG TGTATAAGAGACAGTAGACTTCT GGGTGGCCAAAGAATCA	COI		
VertCOI-VR1d_t1	eDNA Metabarcoding	GTCTCGTGGGCTCGGAGATGTGTA TAAGAGACAGTAGACTTCTGGGT GGCCRAARAAYCA	COI		
VertCOI-VR1i_t1	eDNA Metabarcoding	GTCTCGTGGGCTCGGAGATGTGTA TAAGAGACAGTAGACTTCTGGGT GICCIAAIAAICA	COI		
InvertCOI-fwhF2	eDNA Metabarcoding	TCGTCGGCAGCGTCAGATGTGTAT AAGAGACAGGGDACWGGWTGAAC WGTWTAYCCHCC	COI	95 °C (5 min), followed by 10 cycles at 95 °C (30 s), 64 °C (90 s), 72 °C (30 s) and additional 25 cycles at 95 °C (30 s), at 54 °C (90 s), 72 °C (30 s), and a final extension at 72 °C (10 min)	Vamos, Elbrecht & Leese, 2017; Leese et al., 2020
InvertCOI-EPTDr2n	eDNA Metabarcoding	GTCTCGTGGGCTCGGAGATGTG TATAAGAGACAGCAAACAAATARD GGTATTCGDTY	COI		
MLepF1	eDNA Metabarcoding	TCGTCGGCAGCGTCAGATGTG TATAAGAGACAGGCTTTCCCAC GAATAAATAATA	COI	94 °C (120 s) followed by five cycles at 94 °C (40 s), 45 °C (40 s), 72 °C (60 s); then 35 cycles of 94 °C (40 s), 51 °C (40 s), 72 °C (60 s); and a final extension of 72 °C (5 min)	Hajibabaei et al., 2006
RonMWASPdeg	eDNA Metabarcoding	TCGTCGGCAGCGTCAGATGTGTA TAAGAGACAGGGWTCWCCWGATA TAKCWTTTCC	COI		Hernández-Triana et al., 2014
LepR1	eDNA Metabarcoding	GTCTCGTGGGCTCGGAGATGTGTA TAAGAGACAGTAAACTTCTGGAT GTCCAAAAAATCA	COI		Hebert et al., 2004; Hernández-Triana et al., 2014
HCO2198	eDNA Metabarcoding	GTCTCGTGGGCTCGGAGATGTGT ATAAGAGACAGTAAACTTCAGGGT GACCAAAAAATCA	COI		Folmer et al., 1994
VF2_t1	DNA Barcoding	TGTAAAACGACGGCCAGTCAAC CAACCACAAAGACATTGGCAC	COI	94 °C (120 s), followed by 35 cycles of 94 °C (30 s), 52 °C (40 s), 72 °C (60 s), and a final extension at 72 °C (10 min)	Ivanova et al., 2007; Ward et al., 2005
FishF2_t1	DNA Barcoding	TGTAAAACGACGGCCAGTCGACT AATCATAAAGATATCGGCAC	COI		
FR1d_t1	DNA Barcoding	CAGGAAACAGCTATGACACCT CAGGGTGTCCGAARAAYCARAA	COI		
FishR2_t1	DNA Barcoding	CAGGAAACAGCTATGACACTTCA GGGTGACCGAAGAATCAGAA	COI		

#### Library preparation

PCR amplification for all the samples was performed in triplicate (except stomach content samples) using eDNA extractions and primers targeting the molecular markers 12S (MiFish) (Miya et al., 2015; Miya, Gotoh & Sado, 2020), VertCOI (Hernández-Triana et al., 2017; González et al., 2020), InvertCOI (Leese et al., 2020), and alternative COI primer cocktails for invertebrate identifications in water samples (Folmer et al., 1994; Hebert et al., 2004; Hajibabaei et al., 2006); Hernández-Triana et al., 2014; Milián-García et al., 2020; Milián-García et al., 2023; Milián-García et al., 2025). We performed library preparations from DNA extracts obtained from half- and whole-filter samples from the same site and sampling effort to assess the potential influence of patchy eDNA distribution on the filters.

DNA metabarcoding libraries for the 12S (MiFish) (Miya et al., 2015; Miya, Gotoh & Sado, 2020), VertCOI (Hernández-Triana et al., 2017; González et al., 2020), and InvertCOI (Vamos, Elbrecht & Leese, 2017; Leese et al., 2020) molecular markers were prepared using two PCR reactions. The second PCR was conducted in different rooms and PCR workstations, compared with the first PCR, using dedicated equipment for eDNA library preparation. A multimarker approach (12S and VertCOI) for Canadian fish identification was employed to enhance taxonomic coverage and mitigate blind spots (Morey et al., 2024). A first PCR was performed for each mitochondrial genome (mtDNA) fragment using primers that included the Illumina adaptors (Table 1). Each reaction contained 12.5 μL of 2X KAPA HiFi HotStart Ready Mix (Roche Diagnostics), five μL of each forward and reverse primer (one μM), and 2.5 μL of the DNA extract. The first PCR was conducted in a thermal cycler (Eppendorf Mastercycler) using the PCR cycling conditions cited in the above references. One negative control was included during the amplification. The PCR products were checked on a 1% agarose gel and then purified using a 1:1 NGS magnetic bead (Machery-Nagel) ratio, following the manufacturer’s protocols.

Using de novo synthesized index primers equivalent to the Nextera XT Index Kit (Genomics Facility at the Advanced Analysis Centre, University of Guelph), a second PCR incorporated dual index sets into the Illumina adaptor sequence amplicons. The second PCR was conducted in 50 μL reaction volumes using an Eppendorf Mastercycler thermal cycler, with unique index primer combinations added for each sample. Each reaction contained 25 μL of 2X KAPA HiFi HotStart ReadyMix (Roche Diagnostics), 10 μL of molecular biology-grade water, five μL of each index primer (10 μM), and five μL of the first PCR product after cleanup. The PCR profile consisted of the following steps: 95 °C (3 min), followed by eight cycles of 95 °C (30 s), 55 °C (30 s), and 72 °C (30 s), with a final extension at 72 °C (5 min) (Illumina, 2013). Before sequencing, PCR products were visualized on a 1% agarose gel and purified using a 1:1 NGS magnetic bead (Machery-Nagel) ratio, following the manufacturer’s protocols. Similarly, to minimize contamination, DNA extracts and post-PCR products were treated and processed in separate areas.

#### High-throughput sequencing

Sequencing was performed at the Genomics Facility of the Advanced Analysis Centre (AAC) at the University of Guelph for all analytical samples and controls alike. The sequencing libraries were normalized using the SequalPrep Normalization Kit (Thermo Fisher Scientific, Waltham, USA), pooled, and quantified with the Qubit dsDNA HS assay kit (Thermo Fisher Scientific). The libraries were then checked for fragment size using a Bioanalyzer HS DNA Chip (Agilent). Libraries that passed quality control were sequenced on an Illumina MiSeq System using a MiSeq reagent kit v3 (600 cycles), with 1% of the run reserved per sample. Demultiplexing and adapter trimming of the sequencing reads were performed using the MiSeq Reporter software, resulting in two paired-end raw FASTQ files.

The Illumina reference library preparation guide (Illumina, 2013) was used as a reference for High-Throughput Sequencing (HTS) library preparation. Data analysis for the 12S dataset was conducted as indicated in the MiFish pipeline (http://mitofish.aori.u-tokyo.ac.jp/mifish/), while all COI datasets were analyzed using the Multiplex Barcode Research And Visualization Environment (mBRAVE: http://www.mbrave.net/). An alternative bioinformatics pipeline for data analysis (MetaWorks Porter & Hajibabaei, 2022) was used to confirm species identification and provide statistical support (bootstrap) to the taxonomic identifications.

#### Data analysis

Quality control of each raw data file (FASTQ files) was performed using FastQC (Andrews, 2010) to assess base sequence quality scores, base sequence content, sequence length distribution, sequence duplication levels, overrepresented sequences, and adapter content (http://www.bioinformatics.babraham.ac.uk/projects/fastqc/). Metabarcoding data analysis was conducted using MetaWorks with the following parameterization for read pairing: a Phred quality score cutoff of 20, a minimum overlap of 25 base pairs between reads, a maximum fraction of 0.2 mismatches in the overlap, and a minimum fraction of 0.9 matching overlap. Primer trimming using CUTADAPT (Martin, 2011) consisted of a minimum sequence length of 100, Phred quality score of 20, error rate of 0.1, minimum overlap of 3, and a maximum of 3 N’s. The minimum number of reads per cluster for denoising was 3. Denoising was completed using VSEARCH (Rognes et al., 2016). After denoising, clustering was completed to assign taxonomy.

To determine the fish species present in the samples, we mined data from BOLD and FishBase to create a dataset of species found in Alberta and British Columbia. To mine data from BOLD, we used the bold R package version 1.3.0 (Dubois & Chamberlain, 2023) to retrieve ‘Actinopterygii’ entries from Alberta and British Columbia. To mine data from FishBase, we utilized the rfishbase package in R version 5.0.1 (Boettiger, Lang & Wainwright, 2012) to retrieve freshwater species information for Alberta and British Columbia. We then extracted a list of unique species from the mined entries from BOLD and FishBase to compare against the MetaWorks results. Only MetaWorks results with a bootstrap value of 0.97 or higher were retained and reported for this analysis. A subset of species had low bootstrap values from MetaWorks but was confirmed with alternative pipelines (MiFish). These species/genera were included in all of the following analyses, plots and tables (including *Couesius plumbeus*, *Rhinichthys cataractae*, *Coregonus* spp., *Oncorhynchus* spp.).

To assess the quality of the metabarcoding results, including contamination and tag jumps, and generate rarefaction curves, we used the R package metabaR version 1 (Zinger et al., 2021). MetabaR utilizes information from sampling, sequencing, and preliminary bioinformatic analyses to determine whether any molecular artifacts are present. Datasets for each marker (invertCOI, vertCOI, 12S) were analyzed separately in metabaR using ESV data from MetaWorks. Additionally, plate diagrams were used to input the location of controls. All metabaR input files were created manually, except for the MOTU file, which was created using a custom script.

### Food web characterization (stomach contents)

The Hanner Lab received 33 stomach samples (*C. clupeaformis*) preserved in Longmire’s buffer, which the lab prepared and shipped to store stomach contents following collection. The first batch of stomach samples received had 21 samples, followed by a second batch of 12. Fish for gut analysis were captured in Wood Buffalo National Park waters during domestic fishery activities and sampled before processing for consumption. Lake Whitefish stomachs were harvested from fish captured at sites in the Quatre Fourches Channel near Dog Camp, the Peace River near PAD tributaries confluences as part of the domestic fishery and the ongoing CBM Lake Whitefish tissue contamination and fish health studies (Gibelli et al., 2026). Peace River area (*n* = 5) and Jackfish area (*n* = 10) fish were captured in 4 1/2″stretch mono-filament mesh gill nets set overnight, while some fish were captured in short-set (*e.g.*, 1 hr) floating 4 1/2″mesh gill nets in Chanel des Quatre Fourches. Lake Whitefish collected for the CBM Fish camp had their stomachs inspected as part of the CBM sampling process, harvested, and stored for shipping. Fish captured near Dog Camp at Quatre Fourches had their stomachs harvested and stored for later analysis.

Upon receiving the samples, they were placed in 100% Ethanol to preserve them more effectively. Twenty-four samples were already cut open before subsampling, and most of them had little to no contents inside the stomach. We assumed that the contents were sediments in the buffer and attempted to collect the material for further analysis. We collected three replicates of stomach content (if the stomach was empty, we resorted to scraping the stomach lining) and one stomach tissue sample. We weighed each replicate and tissue sample immediately after collection. We let the ethanol or Longmire’s buffer evaporate from the subsamples before DNA extractions using the Qiagen DNeasy Blood & Tissue Kit (Qiagen).

One of the three replicates sampled from each stomach content was used for eDNA metabarcoding analysis, using the same methodology and molecular markers described above for the eDNA filter samples. Stomach tissue was used to validate species identity *via* DNA barcoding, targeting approximately 658 bp of the cytochrome oxidase I (COI) gene, following the same protocol as for the fin clips described above.

## Results

### Fish community biodiversity monitoring

Successful DNA extractions were obtained from all filters, as evidenced by measurable DNA concentrations in every analytical sample (see details in Table S1). Big Egg Lake (0.182 ng/uL), Embarras (0.034 ng/uL), and Jackfish Site B (0.202 ng/uL) field negatives were the only field negatives that registered a DNA concentration when quantified with the Qubit High Sensitivity (HS) kit, suggesting sporadic contamination in the field during sample collection (Table S1).

**Figure 2:**
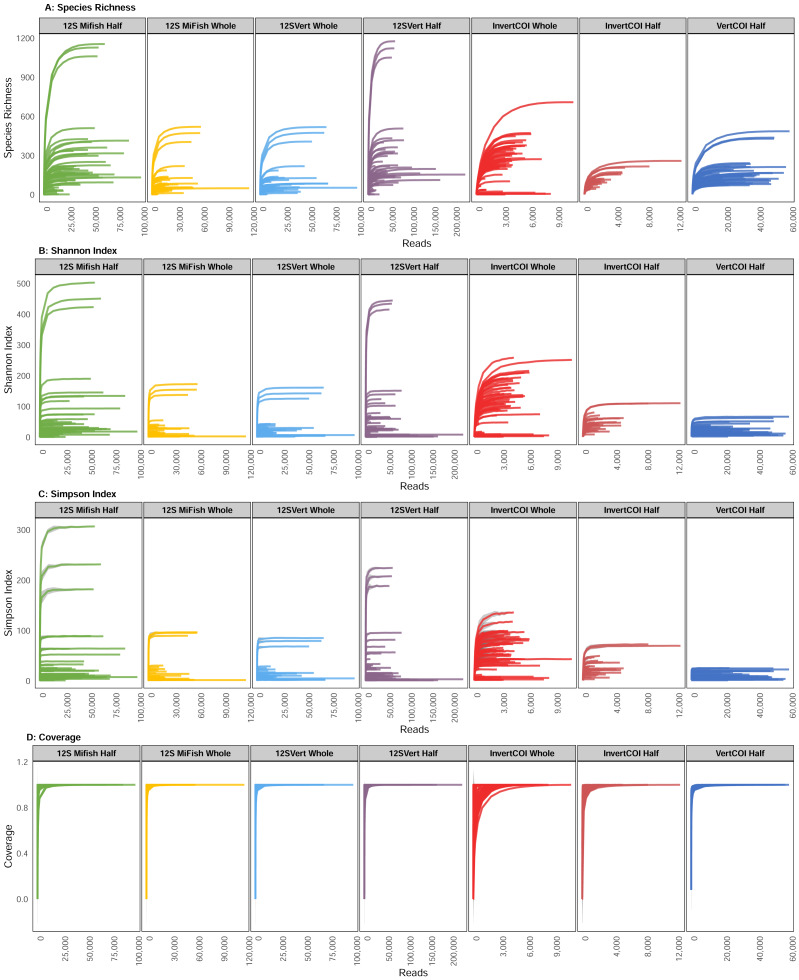
Rarefaction curves for molecular markers for water samples with half and whole filters. Shaded areas represent standard deviation.

Rarefaction curves indicated saturation of species richness for every marker used, regardless of the type of sample processed (half filter or whole filter). The latter suggests that the selected sequencing depth was sufficient to maximize recovery of genetic variants present in the analytical samples for each primer set used in the present study (Fig. 2).

**Figure 3:**
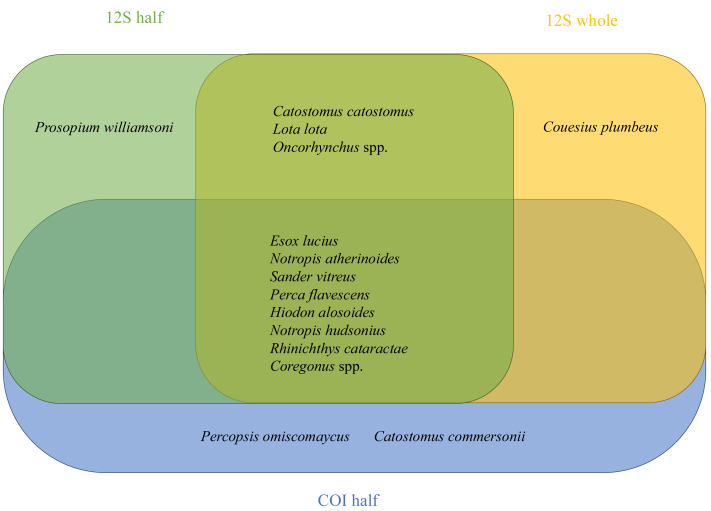
Venn diagram showing specific fish identified when using 12S (half filter and whole filter) and VertCOI (half filter) molecular markers.

Fifteen fish species known to occur in the area, based on historical data from morphological observations or molecular detections reported for the geographic location (records deposited on BOLD and FishBase), were identified using eDNA traces in water samples, as determined by 12S and COI molecular markers (Fig. 3). The lists of fish molecular detections per collection site varied from four (*e.g.*, Big Egg Lake) to twelve (*e.g.*, Jackfish A and Jackfish B) (Table S2). Species molecular identifications were validated using the representative sequence variants detected and multiple alternative DNA reference databases (MiFish, GenBank, and BOLD). Similarly, all additional molecular matches outside the fifteen known fish species detected using eDNA, including those with a low likelihood of occurring in the region, were also checked. Notably, one unexpected match (*Gobio gobio*), in addition to the fifteen previously identified species to inhabit the area, was detected and further evaluated.

**Figure 4:**
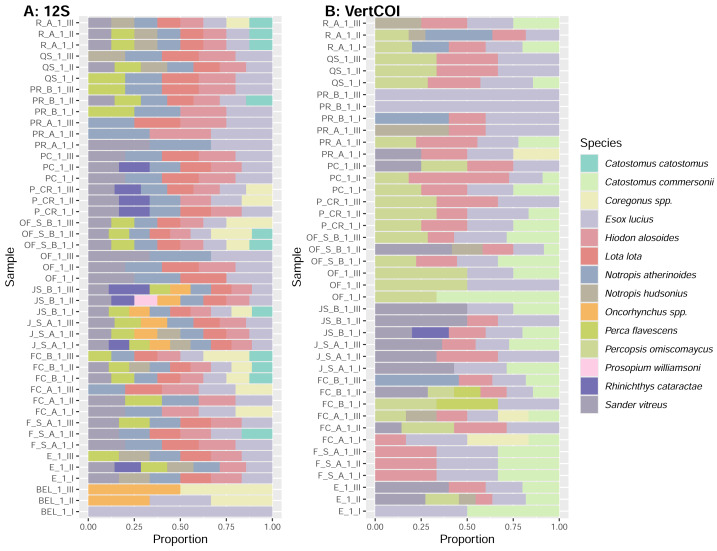
Fish taxonomic diversity in the water samples (half filter) inferred from the 12S (A) and VertCOI (B) primers shown in terms of the proportion of genetic variants per site. The species list per collection site is presented in Table S2.

Overall, eight fish species out of 15 were cross-validated with 12S and COI molecular markers (Fig. 3), suggesting differential resolution and specificity for fish eDNA detection between both markers. As expected, MiFish 12S exhibited higher specificity for fish (Figs. 3 and 4). On the contrary, when using VertCOI, over forty non-fish vertebrate species’ eDNA was detected.

For twelve out of the fourteen fin clips processed in the present study, the sampled fish identity was confirmed based on molecular data. However, two samples (#7 and #14; samples labelled as White Sucker and Goldeye) showed mismatches between molecular and morphological identifications (Table 2). The latter suggests misclassification or, more likely, mislabelling at the sampling location, rather than extensive sample contamination with DNA or tissue from other fish species. Obtained DNA sequences were unambiguously assigned to the reported species instead of the labelled ones. As we received only fin clips in this case, double-checking the morphological ID was no longer possible. To better identify error sources in future collaborations, we suggest retaining images of the specimens from which tissue samples were collected and linking each set of images to each tissue sample, as we consider this good practice moving forward. In total, tissue from six fish species was collected (Table 2). A more intensive sampling targeting all fish species inhabiting the area will be needed to build a representative mock community containing DNA genetic variants from fish occurring in the Peace-Athabasca Delta. The use of such a mock community as a positive control in eDNA metabarcoding analysis will facilitate the evaluation of different molecular marker resolutions (Morey et al., 2024; Kachiprath et al., 2026).

**Table 2:** List of fin clip samples received at the Hanner Laboratory, including fish morphological identifications, molecular identification (DNA barcoding), fragment length sequenced, GenBank Accession numbers, and percentage of identity to the species identified using the GenBank database.

**Sample**	**Sample ID**	**Morphological ID**	**Common name**	**Molecular ID**	**Sequence length**	**GenBank accession #**	**% of identity**	**Location**
1	1-PC-JR	*Coregonus clupeaformis*	Lake Whitefish	*Coregonus clupeaformis*	652	PZ151438	100	Athabasca River at Jackfish Creek
2	2-PC-JR	*Coregonus clupeaformis*	Lake Whitefish	*Coregonus clupeaformis*	675	PZ151439	99.4	Athabasca River at Jackfish Creek
3	3-PC-JR	*Coregonus clupeaformis*	Lake Whitefish	*Coregonus clupeaformis*	652	PZ151440	100	Athabasca River at Jackfish Creek
4	4-PC-PR	*Sander vitreus*	Walleye	*Sander vitreus*	685	PZ151441	99.4	Peace River, Moose Island
5	5-PC-PR	*Sander vitreus*	Walleye	*Sander vitreus*	652	PZ151442	100	Peace River, Moose Island
6	6-PC-PR	*Sander vitreus*	Walleye	*Sander vitreus*	652	PZ151443	100	Peace River, Moose Island
7	7-PC-QF	*Catostomus commersonii*	White Sucker	*Catostomus catostomus*	652	PZ151444	99.9	Quatre Fourches, AB
8	8-PC-QF	*Esox lucius*	Northern Pike	*Esox lucius*	652	PZ151445	100	Quatre Fourches, AB
9	9-PC-QF	*Hiodon alosoides*	Goldeye	*Hiodon alosoides*	648	PZ151446	99.7	Quatre Fourches, AB
10	10-PC-QF	*Esox lucius*	Northern Pike	*Esox lucius*	652	PZ151447	99.9	Quatre Fourches, AB
11	11-PC-QF	*Catostomus commersonii*	White Sucker	*Catostomus commersonii*	684	PZ151448	99.4	Quatre Fourches, AB
12	12-PC-QF	*Hiodon alosoides*	Goldeye	*Hiodon alosoides*	645	PZ151449	99.3	Quatre Fourches, AB
13	13-PC-QF	*Esox lucius*	Northern Pike	*Esox lucius*	652	PZ151450	100	Quatre Fourches, AB
14	14-PC-QF	*Hiodon alosoides*	Goldeye	*Esox lucius*	205	PZ151451	98.8	Quatre Fourches, AB

**Figure 5:**
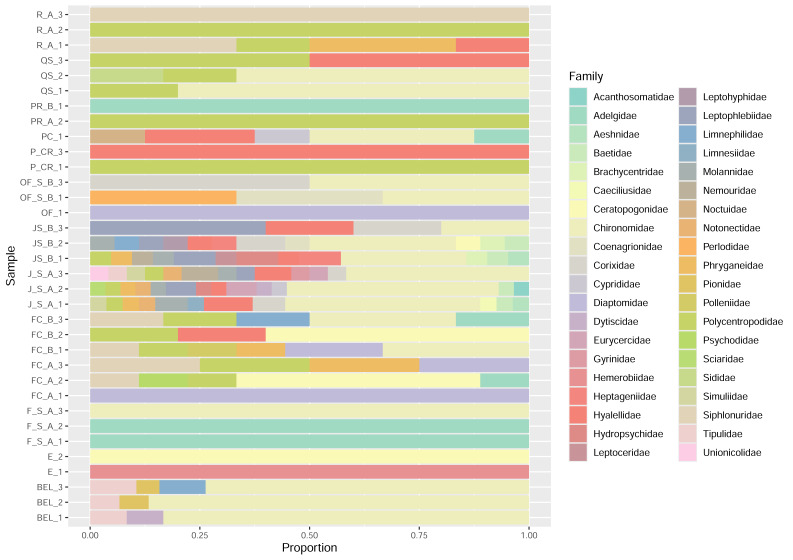
Taxonomic diversity at the family level in the water samples inferred from the InvertCOI primers.

Using the COI molecular marker, filter sample analysis detected 56 families of insects, five families of birds (Anatidae, Rallidae, Phasianidae, Turdidae, Podicipedidae), seven families of mammals (Bovidae, Castoridae, Cervidae, Hominidae, Muridae, Canidae, and Cricetidae) and eight families of fish (Catostomidae, Salmonidae, Esocidae, Hiodontidae, Cyprinidae, Percopsidae, Percidae, Cottidae) along with several families of diatoms, fungi, and algae. The vast majority of samples contained eDNA from Diptera (flies). The majority of flies detected in the analysis were midges (Chironomidae), comprising 60 identified genera. Of those genera, species of *Ablabesmyia, Brillia, Chironomus, Cricotopus, Dicrotendipes, Micropsectra, Orthocladius, Polypedilum, Psectrocladius, Robackia, Tribelos, Tvetenia* and *Xenochironomus* were detected and are known to occur in Alberta (Boerger, 1981; Soluk, 1985; Berg & Hellenthal, 1991). Other families of flies detected included Anthomyiidae, biting midges (Ceratopogonidae), glassworms (Chaoboridae), mosquitoes (Culicidae), meniscus midges (Dixidae), vinegar flies (Drosophilidae), dance flies (Empididae), shore flies (Ephydridae), Lauxaniidae, crane flies (Limoniidae and Tipulidae), house flies (Muscidae), Phoridae, drain flies (Psychodidae), marsh flies (Sciomyzidae), and black flies (Simuliidae). Other insects detected included caddisflies (Trichoptera), mayflies (Ephemeroptera), water boatmen (Corixidae), backswimmers (Notonectidae), stoneflies (Plecoptera), and several families of beetles (Fig. 5). Invertebrate species composition, considering abundance and presence-absence differences among sampled water bodies (river, lake, and creek), was explored using dissimilarity indices (Bray–Curtis and Jaccard). The main differences in species composition were observed between Lake and Creek water bodies (Bray–Curtis index = 0.74), and they also shared a high number of species (Jaccard index = 0.75).

### Food web characterization (stomach contents)

**Figure 6:**
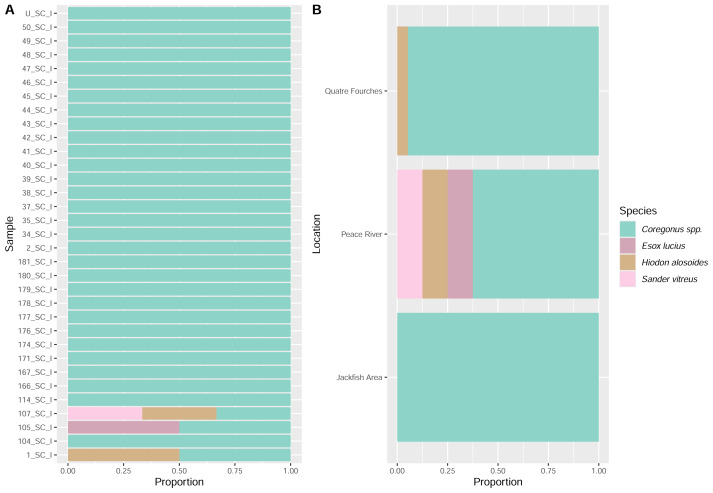
Fish eDNA found in the stomach content (analysis per sample (A) and per collection site (B)) based on the 12S molecular marker.

**Figure 7:**
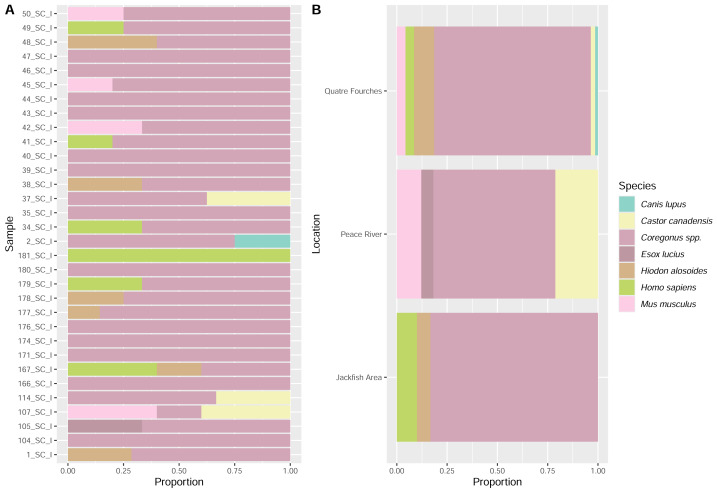
EDNA found in the stomach content (analysis per sample (A) and per collection site (B)) based on VertCOI molecular marker.

Using standard DNA barcoding, genetic material extracted from stomach tissue ratified the molecular identity of all the *Coregonus clupeaformis* (Lake Whitefish) specimens. On the other hand, DNA metabarcoding analysis confirmed the sampled species identity, as *Coregonus* DNA was found in all stomach contents and collection sites (Figs. 6 and 7). DNA from fish species other than *Coregonus* spp. (*E. lucius*, *H. alosoides*, and *S. vitreus*) was also detected in the stomachs through metabarcoding analysis (Figs. 6 and 7). Still, the origin of this DNA (*e.g.*, scales, eggs, or juveniles) remains unknown. However, fish scales were found in the preservation buffer and in several stomachs when subsampling from the stomach contents.

**Figure 8:**
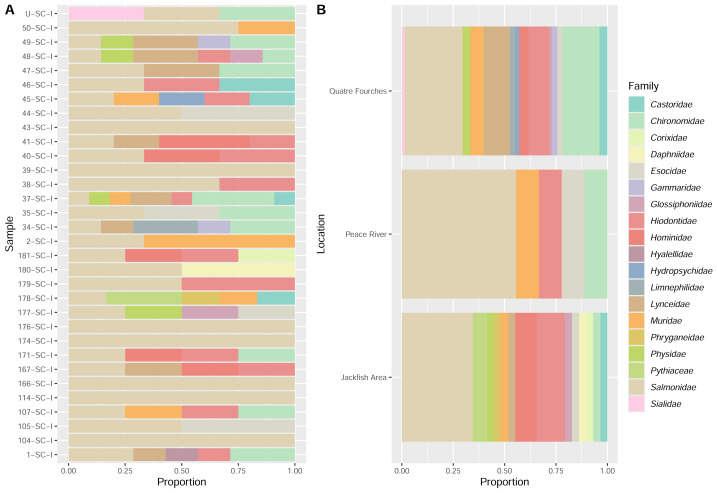
EDNA found in the stomach content (analysis per sample (A) and per collection site (B)) based on InvertCOI molecular marker.

Stomach content analysis also detected several species of Chironomidae (midges) with widespread distributions that encompass the Nearctic region; these species are either recorded in Alberta or likely to be found in Alberta and include *Ablabesmyia americana* (=*Ablabesmyia* (*Ablabesmyia*) *monilis*)), *Dicrotendipes nervosus*, *Tanytarsus mendax*, *Thienmanniella* sp., *Parachironomus* sp. and *Procladius* sp. (Fig. 8) (Boerger, 1981).

All species of Caddisflies (*Hydropsyche* sp. (Nimmo, 1981), *Nemotaulius hostilis* (Berté & Pritchard, 1986) detected in the stomach contents are recorded in Alberta or neighbouring provinces. Both species of Amphipods, *Hyallela azteca* (Podesta & Holmes, 1970) and *Gammarus* sp. (Blais et al., 2003), are recorded in Alberta. Other miscellaneous invertebrates, including *Cymatia americana* (Insect - Water boatmen) (Lapierre et al., 2021), *Helobdella* sp. (Leech) (Davies & Reynoldson, 1975), *Lynceus* sp. (Brachiopod) (Retallack & Clifford, 1980), *Metopobactris prominulus* (spider) (Buckle et al., 2001), *Physella* sp. (Snail) (Johnson et al., 2013) and *Sialis* sp. (Insect, alderfly) (Scudder & Cannings, 2009) are all also either recorded in Alberta or likely to be found in Alberta. The invertebrate species composition was compared in the stomach content among collection sites, using two dissimilarity indices (Jaccard and Bray–Curtis). Overall, the stomach content composition (invertebrates) from Peace River and Quatre Fourches exhibited the highest compositional differences (Bray–Curtis index = 0.78) and also shared a high number of species (Jaccard index = 0.79). A general lack of Mollusc detection was noted, particularly for bivalves and snails, which may be associated with the limitations of the molecular markers used to amplify their DNA or a lack of representation in the DNA reference database, potentially hindering their identification. Notably, snails and clams were noted on the data sheets as being present in the stomach contents. Further efforts will be needed to increase detection probabilities for these taxa.

Terrestrial vertebrate eDNA from a canine (*Canis* sp.), human (*Homo sapiens*) and mouse (*Mus sp.*) was detected in the stomach content analysis (Fig. 7). A genus of leech (*Helobdella* sp.) was also detected in the stomach contents; however, it only feeds on invertebrates (Kutschera et al., 2013), which does not explain the presence of vertebrate DNA (*i.e., via* blood) in the stomach.

## Discussion

### Fish community biodiversity monitoring

In the present study, fifteen fish species known to inhabit the geographic area were identified based on traces of eDNA collected from water samples and a multi-marker approach (12S and COI). Consequently, as implemented here, eDNA-based techniques (*e.g.*, eDNA metabarcoding) have proven advantageous for establishing indigenous community-led biomonitoring programs, owing to the non-invasiveness and straightforward nature of sample collection relative to traditional capture-based monitoring approaches (*e.g.*, electrofishing, gillnetting). The harmless simplicity of eDNA sample collection has made these methods highly appealing for citizen science involvement, which remains one of the key promises of eDNA use in tackling global biodiversity challenges (Altermatt et al., 2025). Collectively, the dual marker approach enabled a greater number of fish eDNA detections. But differential recovery rates in taxonomic richness were observed between COI and 12S, with the 12S being the most effective for eDNA fish detection. The latter was expected, given the versatility of the COI cocktail primers used, not exclusively designed for fish detection. Even when this is the case, 12S has demonstrated the ability to recover higher fish diversity than COI (Zhang, Zhao & Yao, 2020). An in-depth study of potential primer blind spots for each marker used can be derived from a fish mock community evaluation (Morey et al., 2024; Kachiprath et al., 2026), representing the fish diversity of the geographic area. This was not possible in the current study, as we were limited in the number of fish species whose DNA was collected from fin clips (only six species).

For the study area, the total number of species confirmed based on eDNA traces in water samples was fewer than the total historical number of fish species detected using traditional methods. For example, through electrofishing and minnow-trapping efforts for other research projects, approximately 28 fish species have already been identified in the area (Bruce Maclean, pers. obs., 2026). Notably, a single eDNA sampling event, such as the one conducted in our study, is not intended to capture every fish that has historically inhabited or been caught in the sampled water bodies. On the contrary, it provides a snapshot of what is present near the site of water sampling at a given moment in time. Similarly, when sampling *via* traditional methods such as electrofishing or netting at the same site at different times, there is not necessarily the expectation to catch the same fish species each time, as their presence also varies across time and space. In our experimental context, processing three technical replicates of a half-filter sample per site was sufficient to detect the highest number of fish eDNA, compared with whole-filter analysis. When subdividing the filter, patchy eDNA distribution on the filter has been suggested as a potential factor in differential detection rates. This is particularly critical for rare or low-abundance taxa, for which eDNA might be present in low amounts. Consequently, avoiding splitting the sample has been recommended for those cases (Milián-García et al., 2023). However, this was not an apparent factor in our study. Using filter fragments can reduce the time and cost per sample processed (*e.g.*, by halving the number of DNA extractions), while keeping voucher samples for future analysis. In any case, future studies would benefit from increasing biological replication while keeping at least the same technical replication level, without subdividing the sample, to maximize detection probabilities per sampling effort.

Importantly, no method (including eDNA-based approaches) is without limitations, and targeting short eDNA fragments (*e.g.*, less than 220 bp) to account for environmental DNA degradation compromises resolution and species detection rates. The latter implies extensive geographic curation, in-depth marker resolution analysis, and evaluation of bioinformatic outputs to minimize false positives without increasing false-negative rates. High false-positive and false-negative rates, as well as a lack of thorough discussion about the limitations of eDNA-based methods, can impact the uptake and reliability of these molecular tools.

The collected eDNA traces signal what was in proximity to the water sample within a few hours to days of sampling, just as finding land animal tracks (*e.g.*, buffalo) can indicate that the animal passed through an area, even if it was not necessarily visible on the landscape at that moment. Also, just as animal tracks fade over time, so too does the eDNA signal after a fish has passed through. The fade of the eDNA signal can be caused by a myriad of factors, such as higher temperatures (*e.g.*, >50 °C), high UV light exposure, microbial load and its community composition, and extracellular nucleases (Lamb et al., 2022). It is also expected that longer eDNA fragments will degrade faster than shorter fragments (less than 220 bp), which is a key reason the eDNA community advocates for shorter target fragments. However, even if shorter DNA sequences are expected to degrade more slowly, given more chances to detect the molecular signal in the environment, they can also compromise marker resolution. The latter can limit the researcher’s capacity to classify unknown sequences to lower taxonomic ranks when compared against DNA reference libraries. This has been evidenced here in the case of *Coregonus* or *Esox* species.

In that regard, within the present study’s context, representative 12S sequences linked to *Esox flaviae* are 99.4% similar to *Esox lucius* sequences deposited in the GenBank database (MH918123.1). It indicates the close genetic identity of congeneric species within the genus *Esox,* suggesting that the marker sequence lacks species-level resolution. Consequently, *Esox flaviae* (a European species of pike) hits were inconsistent across different molecular markers and alternative bioinformatics pipelines (*e.g.*, MiFish) for data analysis, rendering it a low-confidence record. As a result, any *E. flaviae* hits were considered to represent *Esox* sp. instead.

The use of multiple bioinformatic pipelines reveals that, for some species, a high percentage of identity does not always correlate with high statistical support. Other marker sequences showed low resolution at the species level. For example, *Coregonus artedi* had a percent identity of 99.41 but a bootstrap value that corresponds to statistical support below the initial cut-off of 0.97 (Table S2). The representative sequence corresponding to *C. artedi* was 100% identical to other *Coregonus* species deposited in GenBank, including *C. artedi*. This pattern also holds for other species with a high percentage of identity and low bootstrap support, such as *Oncorhynchus mykiss,* where there is a low resolution at the species level. Additionally, representative sequences for *Percopsis omiscomaycus* were 100% identical to those of *P. transmontana*, a species not present in the study area. This highlights the importance of validating results from multiple bioinformatic pipelines and the need for extensive and careful geographic curation.

In general, there is a trade-off between DNA fragment length and molecular-based species resolution. Longer fragments may increase resolution, but at the cost of reducing amplification success when working with eDNA, due to high levels of degradation in the environment. The longest amplified fragment for the current eDNA metabarcoding analysis targeted 220 bp (12S). At this level, a lack of resolution was observed within members of the genus *Coregonus*, with *C. artedi* and *C. clupeaformis* exhibiting high molecular similarity over the short DNA fragments used. On the contrary, unambiguous classifications for *C. clupeaformis* were obtained when targeting 658 bp of the COI from DNA extracted from the stomach samples or fin clips, using standard DNA barcoding.

The representative gudgeon (*Gobio gobio*) sequence detected in the current study (Table S4) was 100% identical to other gudgeon sequences deposited in GenBank (*e.g.*, LC468875.1). We were unable to find evidence of the gudgeon being previously detected in North America; there is no evidence of *G. gobio* in any minnow sampling efforts completed by CBM (Bruce Maclean & unpublished data CBM, pers. obs., 2026), ECCC (unpublished data, Craig Hebert) or the province of Alberta (AB; Dr. Mark Poesch, pers. comm., 2026). Significantly, a single eDNA-based detection of an invasive alien species not previously reported in the area should not trigger a policy response for removal, but rather the deployment of alternative methods to confirm its presence (Altermatt et al., 2025). Further work would be required to determine whether this is a marker-related issue (*i.e.,* primer mismatch/misclassification), a DNA reference library issue, contamination, or if it signals the first documented invasion of the PAD by the gudgeon. It is essential to highlight that *Gobio gobio* is not a species we work with at the Hanner Laboratory. Hence, the risk of cross-contamination with the species’ eDNA is low in our laboratory. However, false-positive detections from eDNA metabarcoding data are a known limitation and should be carefully evaluated. Although eDNA’s sensitivity is widely recognized and used to infer a taxon’s presence from its environmental signal, this indirect inference is sometimes also considered a major weakness (Altermatt et al., 2025). To detect and reduce false-positive rates, we included regular field and laboratory negative controls (*e.g.*, extraction and PCR controls) and tracked potential contamination at every step of our workflow. We found no evidence of cross-contamination (sporadic or systemic) with any alternative source of gudgeon eDNA in any step of the analysis. Interestingly, the species has ornamental value, and some retailers in Canada, including within Alberta (Gobio Gudgeon (Gobio Gobio) | Amazonia Aquatics Canada), commercialize it. Consequently, overlooked eDNA contamination sources (*e.g.*, runoff of genetic material from aquariums into surrounding habitats) resulting from human activities may contribute to false-positive detections (Zhan, 2025). Recurrent sampling and deployment of alternative molecular identification approaches, particularly targeted detection methods (*e.g.*, ddPCR or qPCR), would be necessary to untangle the cause of this detection. Notably, the United States Fish and Wildlife Service (USFWS) lists the species as having high invasive potential (U.S. FWS, 2018); therefore, further investigation will be necessary to clarify this case. Importantly, the PAD is a protected area (part of WBNP, a Ramsar and UNESCO World Heritage site), implying that strict regulations and monitoring make unauthorized species introductions less likely. The latter is critical, as the gudgeon introduction in the PAD would require deliberate or accidental human action (*e.g.*, upstream areas (Fort McMurray) where aquarium releases are possible), which is neither documented nor suspected in this region. If the introduction is confirmed, accidentally or deliberately, it should trigger a management response from Parks Canada, which is responsible for protecting the ecological integrity of the waters within the park and controlling potential ecological harms caused by an invasive alien species.

At least 28 fish species are known to inhabit the PAD, with fifteen of them successfully identified in the present pilot study *via* eDNA metabarcoding (Table S2 and Fig. 3). More sampling effort (spatial and temporal) and improvements in molecular markers will be needed to increase the detection probability of non-detected species occurring in the area (*e.g.*, *Salvelinus namaycush* [Lake trout] or *Coregonus clupeaformis* [Lake Whitefish]). Lake trout and whitefish are also more benthic-oriented due to their feeding behaviour and a seasonal preference for cooler water; therefore, their DNA is less likely to be captured using eDNA, as samples are typically collected near the surface. Seasonal avoidance of warmer water temperatures might have biased some results against their detection. Consequently, eDNA detection for specific fish species may be hindered if samples are taken only at the surface, when fish prefer cooler lake waters or in moving waters, such as rivers (Pont et al., 2018; Littlefair et al., 2021; Fukumori et al., 2024). A depth-integrated water sampler can help address this issue. It is essential to note that our results are based on a single sample taken from the surface water at each sampling location. In the case of the genus *Coregonus*, we also observed an overall lack of resolution with the short molecular markers used in the eDNA metabarcoding approach for distinguishing species within the genus. Only species detected as matching the fish species list retrieved for the study area and cross-validated by multiple bioinformatics pipelines were reported. These were the only cases where the lower bootstrap below the initially established cut-off (0.97) was accepted. The latter included specific species, such as *Rhinichthys cataractae*, that would have otherwise not been reported, not necessarily representing a true absence. Further studies will benefit from extending 12S amplicon size, as it has already been demonstrated to improve taxonomic resolution (Yates et al., 2025). In the current pilot study, we explored three bioinformatics pipelines (MetaWorks, MiFish, and mBRAVE) for analyzing metabarcoding data. MiFish and mBRAVE are web-based, marker-specific pipelines for analyzing 12S and COI metabarcoding data, respectively. They both allow rapid metabarcoding data processing, outputting sequence-based molecular taxonomic classification from sequencing data. Their user-friendly nature, graphical user interface (GUI), and ability to run from any operating system with minimal bioinformatic background make them appealing for citizen-science or community-based monitoring applications. They then enable more straightforward data analysis checks by third parties and collaborators with minimal training or bioinformatic infrastructure required. However, they offer limited scalability capacity and control over the reference libraries used for taxonomic assignments. Alternatively, MetaWorks is a scalable Linux-based command-line pipeline, designed to process multiple marker datasets within a single environment (Porter & Hajibabaei, 2022). It also uses an alternative sequence-composition-based taxonomic assignment method (naive Bayes classifier). Using multiple bioinformatics pipelines with different taxonomic classifiers to validate taxa identifications is strongly recommended. Especially because differential classifiers have demonstrated variable performance, which not only varies by classifier but also by marker (Bayer et al., 2025). If eDNA were accepted as part of the Integrated Resource Management Plans (IRMP) Fish Abundance and Distribution Indicator, we also recommend updating the methodology to incorporate eDNA sampling alongside traditional netting. This would help determine which species are captured *versus* those that are or were nearby when the netting occurred. This would provide a more complete species presence /absence detection by site.

### Food web characterization (stomach contents)

Lake Whitefish (*C. clupeaformis*) is a primarily benthic feeder (Pothoven & Nalepa, 2006; Pothoven & Madenjian, 2008). Although numerous studies on whitefish diets in the Great Lakes have been conducted (Pothoven & Nalepa, 2006; Pothoven & Madenjian, 2008; Nalepa, Pothoven & Fanslow, 2009), there is a lack of recent published information describing whitefish diets in the PAD. A study in the nearby lower Slave River (Northwest Territories) recorded Lake Whitefish to feed primarily on ostracods during the spring, adult corixids (water boatmen) during the fall and a mixture of corixids, trichopteran larvae (caddisflies) and gastropods as primary food sources in the summer (Retallack & Clifford, 1980). A study of the Athabasca and Clearwater rivers found that Lake Whitefish feed mostly on adult corixids before spawning and whitefish eggs after spawning (Lapierre et al., 2021). Another study found that mostly Corixidae, midges (Chironomidae), and caddisfly larvae, along with fish eggs, clams (*Sphaerium* spp.), and whirligig beetles (Gyrinidae), were present in the Athabasca River, associated with whitefish (Podesta & Holmes, 1970). In a study of Great Bear Lake (Northwest Territories), Johnson (1975) (Johnson, 1975) found the whitefish diet to be composed mostly of Sphaeriid clams, *Pontoporeia* and *Gammarus*.

In this case, the collection of stomachs and reduction of diet information were intended to complement the research that the CBM program is conducting on Lake Whitefish contamination in the domestic fishery. Knowing what Lake Whitefish were eating may assist with ongoing research on Hg, metals and PAC/PAH levels in tissues. Local CBM and the Government of AB also collect isotope samples and conduct comprehensive analyses on Lake Whitefish, indicating trophic levels of feeding. Furthermore, combined with the recently collected genetic stock information (Gibelli et al., 2026), detailed feeding behaviour and its possible differences could be thoroughly examined for the separate stocks relative to contamination levels. The pilot research completed here demonstrated that a purposefully designed and integrated research program could provide valuable insights into feeding behaviour and contaminant uptake vectors that may exist for fish species or separate stocks of concern. The current study’s stomach content analysis detected nine of the 19 diet items listed in Table 3. It detected all of the primary diet items listed in the sources above, except Sphaeriids and *Pontoporeia.* Whitefish DNA (and other fish DNA) was detected in the stomach content samples; however, it is unknown whether this DNA originated from scales, eggs, juveniles, or adults of the same species. Human DNA presence was deemed due to sample contamination during sampling or filtering. Most of the stomach samples received had already been opened, and the stomach contents had been explored, which requires a higher level of manipulation and consequently increases the risk of cross-contamination. Depending on the sample collection and handling conditions (*e.g.*, when transferring from Whirl-Pak to bottled solutions in the Parks Canada warehouse), DNA from other vertebrates (*e.g.*, dog, mouse) could have been introduced into the samples through contamination. In the present study, only versatile primers were used, which, as seen, can cross-amplify a wide range of taxa, including some considered contaminants in our particular experimental context. Certainly, any non-prey DNA can compete and influence prey DNA detection. To minimize this competition and enhance the amplification of prey DNA in the future, the use of blocking primers could be beneficial, as empirical evidence demonstrates (Zhang, Zhao & Yao, 2020). While collecting subsamples from the stomachs, we also observed whole or partial specimens of Corixidae, snail shells, bivalve shells, fish scales and insect wings. This poses a clear limitation of the molecular markers used to identify molluscs, in particular, which were represented in considerable abundance. To increase the detection probability of these taxa moving forward, considering specific primers for mollusc eDNA metabarcoding identification would be advisable (Shi et al., 2024). Analysis of the water samples revealed eDNA from an additional four diet items not recovered from the stomach contents.

**Table 3:** Whitefish diet based on sources from the Athabasca Delta and surrounding area, including taxon name, reference and whether the diet item was detected in the current stomach content analysis.

**Taxon**	**Common name**	**Source(s)**	**Detected in the Stomach contents**	**Detected in the water samples**
Amphipoda	Amphipods	Little et al., 1998, Johnson, 1975	YES	NO
Ceratopogonidae	Biting midges	Little et al., 1998	NO	YES
Chironomidae	Midges	Little et al., 1998, McCart, Mann & Jones, 1978, Johnson, 1975	YES	YES
Corixidae	Water boatmen	Little et al., 1998, Jones et al., 1978, McCart, Mann & Jones, 1978	YES	YES
Cottidae	Sculpins	Johnson, 1975	NO	YES
Diptera	Flies	Little et al., 1998	YES	YES
Dytiscidae	Diving beetles	Little et al., 1998	NO	YES
Ephemeroptera	Mayflies	Little et al., 1998	YES	YES
*Gammarus*	Amphipod	Johnson, 1975	YES	NO
Gasterosteidae	Sticklebacks	Little et al., 1998	NO	NO
Gastropoda	Snails and slugs	Little et al., 1998	YES	NO
Gyridinae	Whirligig beetle	McCart, Mann & Jones, 1978	NO	YES
*Mysis*	Mysid crustacean	Johnson, 1975	NO	NO
Ostracoda	Ostracods	Little et al., 1998	YES	NO
*Pontoporeia*	Amphipod	Johnson, 1975	NO	NO
*Pungitus pungitus*	Stickleback	Johnson, 1975	NO	NO
*Sphaerium*	Clams	McCart, Mann & Jones, 1978, Johnson, 1975	NO	NO
Trichoptera	Caddisflies	Little et al., 1998, McCart, Mann & Jones, 1978, Johnson, 1975	YES	YES
Whitefish eggs, Fish eggs		Jones et al., McCart, Mann & Jones, 1978	Possible	Possible

## Conclusions

eDNA metabarcoding provided robust detection of fish and other vertebrates, as well as invertebrate species, confirming its potential for biomonitoring and food web characterization in the PAD. Differences in the performance of each molecular marker underscore the need for comprehensive reference sequence libraries—building a reference database with molecular and taxonomic data for the fish species captured in the Peace-Athabasca Delta will benefit future molecular-based identifications.

An assessment of potential taxonomic blind spots arising from the use of molecular markers (*e.g.*, testing against mock communities) is necessary. Future comparisons of feeding behaviour by collection sites will need to include a balanced sampling design. Given the unequal sample sizes, no comparison was completed in the current study. As this research was conducted as a pilot project, findings were limited, and many aspects could be modified for increased success in the future. To better understand fish diversity across the PAD, a wider variety of sampling sites and a higher number of biological replicates, including sampling at different depths, should be considered. Continued and increased collaboration with local ecologists, biologists, and Indigenous Communities is encouraged to help validate eDNA results found in samples from the PAD, as there is very little recent literature and information concerning community structure and diversity in the PAD. If eDNA is adopted as part of the IRMP Fish Abundance and Distribution Indicator, we recommend integrating eDNA sampling with traditional netting to compare captured species with those present nearby during sampling, thereby providing a more comprehensive site-level assessment of species presence and absence.

## Supplemental Information

10.7717/peerj.21341/supp-1 Supplemental Information 1 Collection data sheets and fish health assessment protocols

10.7717/peerj.21341/supp-2 Supplemental Information 2 DNA quantification measurements conducted for the eDNA extracts obtained from each sample and field negative controls (NC)

10.7717/peerj.21341/supp-3 Supplemental Information 3 Species list per collection site generated from eDNA detection in the sampled water bodies using the MetaWorks pipeline (12S half and whole filter analysis) and VertCOI. Matches in fish detection based on 12S whole filter and the COI (VertCOI) molecular m

10.7717/peerj.21341/supp-4 Supplemental Information 4 Total number of raw reads obtained per sample

10.7717/peerj.21341/supp-5 Supplemental Information 5 Top hit from MiFish pipeline for *Gobio gobio*, including representative sequence, percentage of identity, and number of reads found in its associated sample

10.7717/peerj.21341/supp-6 Supplemental Information 6 Interactive map showing the fish species detected based on eDNA

## Additional Information and Declarations

### Data Availability

The following information was supplied regarding data availability: The raw sequencing data (eDNA Metabarcoding) are available in the Sequence Read Archive (SRA) BioProject PRJNA1437261 and PZ151438–PZ151451.
