# Supplementary material for: Community-based eDNA metabarcoding for monitoring fish biodiversity and food webs in the Peace-Athabasca Delta
Source: PeerJ. 2026 May 22;14:e21341. doi: 10.7717/peerj.21341 (PMC13200620; doi:10.7717/peerj.21341)
Supplement: Supplemental Information 1 [file peerj-14-21341-s001.docx]

**APPENDIX 1: Collection Data Sheets, Extended Methods related to the molecular analysis, and fish health assessment protocols and output figures.**

Table of Contents

[DNA Data Sheets 2](#_Toc216027251)

[Fish health assessment: Sampling protocols and Figures 30](#_Toc216027252)

[Fish health assessment (gill, liver and muscle tissue samples) 30](#_Toc216027253)

## DNA Data Sheets

eDNA Collection Data Sheet Instructions

GEN-FISH, University of Windsor, Windsor, ON

Please complete one eDNA Collection Data Sheet per sample site

ALWAYS TAKE eDNA WATER SAMPLES FIRST BEFORE DEPLOYING OR USING CONVENTIONAL SURVEY EQUIPMENT

*Traditional survey equipment can introduce DNA that can contaminate environmental samples

*Do NOT take samples in close proximity to traditional survey equipment

ALWAYS SAMPLE DOWNSTREAM SITES FIRST IF COLLECTING FROM MULTIPLE SITES IN SAME LOTIC SYSTEM

*Collecting samples at upstream locations can introduce contaminating DNA into the environment that can be carried downstream and subsequently collected

CHILL AND/OR FREEZE FILTERS ASAP

*Immediately place filters in chilled storage container for transportation after filtering (e.g. on freezer packs cleaned with bleach), and place in a *clean* freezer (-20C) as soon as logistically possible

**Colour Code:**

- **Critical Information –** sample will be compromised without this data
- **Important Information –** important data for modelling/biological inferences
- **Informative, but not crucial –** data that would improve modelling, but not essential

**Site Identification and Team Details:**

- **Project Name**
- **Site Code:** Use three initials of principal member of field crew, last two numbers of year, and three numbers representing collection’s chronological place in that year.

***Example:*** *FBC-95-001* or *FBC-95-Jun-001*

- **Collector:** Record name of the member of the field crew conducting sampling.

***Example:*** *F Cross, C Darwin, and E Ricketts* or *NE Mandrak and ichthyology class.*

- **Recorder:** Record name of the member of the field crew completing most or all of field sheet.
- **Date:** (dd/mm/yy)
- **Arrival Time, Departure Time:** Record the time when arrived at, and left, field site, using the

24-hour system (i.e., “military time”).

***Example:*** *Start Time: 1300h Stop Time: 1500h*

- **Start Time, Stop Time:** Record the time when fieldwork began and ended at the site, using the

24-hour system (i.e., “military time”).

***Example:*** *Start Time: 1300h Stop Time: 1500h*

- **Waterbody Name:** Do not abbreviate principal name of river, creek, wetland, lake, etc. Compass points used as adjectives of the principal name may be abbreviated. The following
- abbreviations for the types of waterbody may be used, but all others must be spelled out: R. = River; Cr. = Creek; Res. = Reservoir.

***Example:*** *Solomon River* or *N. Fork Solomon R.* or *Wilson Reservoir* or *Cedar Bluff Res.*

- **Latitude, Longitude** (dd.ddddd, -dd.ddddd): take using GPS as close to sampling location as possible.
- **Narrative Locality Description:** Give the distance north or south and then east or west from a sizable town (e.g., county seat) or some other prominent feature nearby to the study site.

***Example:*** *26 km and 8 km E of Hays on FAS 235 (Saline River Road) E of US Hwy 183.*

- **For Lotic Systems Only:**
- **Paired Site:** Can this sampled be ‘paired’ with a corresponding upstream/downstream site on the same system, i.e. was collected immediately adjacent (within 100-200 m upstream or downstream) of another site? Note that sites can be paired with both a downstream and upstream adjacent site.
- **Upstream | Downstream:** If paired, is this the ‘upstream’ or ‘downstream’ sample? Circle one or both (if sites are both immediately upstream or downstream)
- **Paired Site Code:** Record the site code of any other ‘paired’ samples

**eDNA Sampling Method:**

- **Gear Type** – OSMOS, manual, other (be specific)
- **When Filtered?** – date (dd/mm/yy); military time (xx:xx)
- **Where Filtered?** - onsite, lab, other (e.g. motel)
- **Time Until Sample Frozen** – Record how long (in hours) between when samples were collected and placed in a freezer for storage.

*Note: samples should be frozen as soon as logistically possible

- **Preservation Method** – how samples were preserved (RNAlater or silica beads).
- RNAlater is ***required*** for preservation if study objectives include analysis of RNA from environmental samples (e.g. microbial community transcriptome, eRNA, etc.)
- Silica beads are a preferred option when *only* analysis of DNA data is required – silica beads produce high-quality eDNA and are a ‘chemistry-free’ preservation method, reducing the impact of preservation on downstream processing (e.g. extraction, quantification, etc.) and improving standardization. Note, however, that silica bead preservation *will degrade any RNA collected in the sample* – you will not be able to recover eRNA or microbial RNA at a later time-point
  - Note: Dry filter on manifold (i.e. maintain suction after water has been completely filtered) for 30 seconds to minimize the amount of water retained on filter prior to storage with silica beads. Silica beads will become saturated and ineffective if exposed to too much moisture. Note that beads change colour when saturated – if complete saturation of beads occurs, exchange with new, dry beads.
- **Sample No. –** Record how many replicates were created (3 replicates are typically requested)
- **Volume Filtered** (ml) – volume of water filtered
- **Pre-filter** – was water sample pre-filtered?
- **Was a blank created? –** Yes or No. Field blanks are required every 3 sampling sites if sampling from the same water body or every time you move by vehicle to sample a new location.
- **Filtering Time** (sec) – time taken to filter sample
- **Filtering Pressure** – if known, e.g. OSMOS readout
- **Filter Pore Size -** Record the filters being used, as well as material (typically 1.5um GF filters)
- **Other:**  Record any other observations you think are important

**If Lentic:**

**Lake Site Dimensions:**

- **MAX Site Depth (m)**: depth at exact sample location using meter stick, graduated line, handheld sonar.
- **Distance from Shore Sampled (m)**: distance sample taken from shore.
- **Depth Sampled (m)**: depth at which sample taken using meter stick, graduated line, etc.

**Habitat Classification:**

- Circle the general habitat classification/type that best describes habitat from which sample was collected.

**Thermocline:**

- Circle whether the lake is stratified (yes | no | unknown) and, if yes, whether the sample was collected above the thermocline (y | n)
- If possible, provide an estimate of the thermocline depth (e.g. from a temperature/depth profile estimated using a YSI probe)

**If Lotic:**

*Note: If study objectives include estimating eDNA concentrations/quantity, then estimating discharge is **critically** important. The ‘Colour Code’ for the ‘**Depth Measurements**’ and ‘**Stream Velocity Measurements**’ data therefore changes from (•) to (•)

**Stream Site Dimensions:**

- **Stream Width (m)**: use laser range finder, tape measure.
- **Habitat Type (Riffle | Run| Pool)**: Circle habitat type describing to sample collection location
- **Hydraulic Head** **(mm)**: distance water rises above the surface when a meter stick is placed upright on top of substrate in the thalweg (middle of stream).
- **Distance from Shore Sampled (m)**: distance sample taken from shore.
- **Depth Sampled (m)**: depth at which sample taken using meter stick, graduated line.
- **MAX Site Depth (m)**: depth at exact sample location using meter stick, graduated line, handheld sonar.
- **Depth Measurements:** If possible, take five depth measurements along horizontal stream transect equidistant from each other and shore line, with middle estimate taken mid-stream:

**Bank**

**Bank**

**Stream Flow:**

- **Stream Flow (None | Slow | Medium | Fast)**: estimated if not measured.
- **Flow Meter Used**: e.g. Swoffer, orange, etc. (if measured).
- **Stream Velocity Measurements:** Minimum of one stream velocity measurement in stream thalweg. If possible, take three measurements along horizontal stream transect equidistant from each other and shore line, with middle estimate taken mid-stream

**Bank**

**Bank**

- **Flow Relative to Typical Levels**: are stream levels low, typical, or high *for that season* (circle one) – record only if familiar with historical site conditions

**For all Sites (both Lentic and Lotic):**

**Site Characteristics:**

Measure water-quality parameters at exact location and depth of water sample. Repeat measurements at same depth 1 m to the left (or upstream in flowing waters) and 1 m to the right (or downstream). If measurements vary by more than 5%, confirm that equipment is working properly and repeat measurements. Record instrument used.

- **Water Temperature, Air Temperature:** Record the values for each.
- **Conductivity:** Record the value from a conductivity meter.

***Example:*** *540 μS/cm or μmhos/cm at 18^o^C or 25^o^C*

- **pH:** Record the value to the nearest 0.1 standard pH unit.
- **Dissolved Oxygen:** Record the value, if determined.
- **Secchi Disk/Tube (0.00 m)**: Circle gear used and record value determined.
- **Turbidity:** Record the value from a turbidimeter (values other than NTU should be noted).
- **Other Chemical Measurements:** List other chemical attributes tested, if any, and their values and units.

**Substrate:**

Use modified Wentworth Scale: bedrock (>4000 mm); boulder (256-4000 mm); cobble (64-256 mm, rounded); rubble (64-256 mm, angular); pebble (16-64 mm); gravel (2-16 mm); sand (0.06-2 mm, gritty); organic debris (coarse); silt (<0.06 mm); clay.

Describe in one of three ways:

- Estimate dominant substrate by eye or touch *in situ*.
- Estimate % of each major substrate *in situ*.
- Take grab sample, place in bottom of 5 l bucket, shake to distribute evenly, and photograph. Estimate % of each major substrate.

**Biological Components:**

Estimate of each vegetation type or simply dominant vegetation.

- **Aquatic Vegetation**: record % of none, filamentous algae, submergent, floating, emergent. Can use specific names if known.
- **Riparian Vegetation**: record % of none, grasses, shrubs, trees. Can use specific names if known.
- **Aquatic Animals**: record any animals observed (e.g. snails, leeches).

**Floodplain Components:**

- **Floodplain Use**: List primary uses of the area near site. Use the following categories:

*forest – grassland – cropland – residential – industrial/commercial.*

- **Bank (% slope)**: Estimate degree of bank slope, 0-90^o^.
- **Channel Cover (%)**: Estimate % of site for which a canopy of trees or other vegetation extends over the water.

**Weather:**

- Record the general conditions during the visit and in past 24 h.
- **Wind Speed**: Provide only if have instrument, e.g. Kestrel.
- **Wind Direction**: Use ordinal directions to two levels (e.g. NE).

**Photographs:**

Please take a digital photograph of each sampling site. Record photograph file numbers for each category.

- Site
- Aquatic/Terrestrial Habitats
- Algae, if present
- 10 cm water bottom of 5 gal bucket
- Substrate bottom of 5 gal bucket
- Filter after filtering
- Completed field sheets

*Lentic waterbodies

eDNA Collection Data Sheet - GEN-FISH, University of Windsor, Windsor, ON

| Project Name |  | |
| --- | --- | --- |
| **Site Code** |  | |
| **Collector:** | | **Recorder:** |

| Date (ddmmyy) |  | | |
| --- | --- | --- | --- |
| Arrival Time | : (24h) | Departure Time | : (24h) |
| Sampling Start Time : (24h) | | Sampling Stop Time : (24h) | |

**Sample Location**

| Waterbody Name |  | | |
| --- | --- | --- | --- |
| Latitude (dd.ddddd°) |  | Longitude  (-d.ddddd°) |  |
| **Narrative Locality:** | | | |

**eDNA Sampling Method**

| Gear Type | When Filtered?  (ddmmyy; xx:xx) |
| --- | --- |
| Where Filtered?  (onsite/lab/other) | Time until samples  frozen (hrs): |
| Filter Preservation  Method: | Other: |

| **Sample No.** | **Volume (ml)** | **Pre-Filter (Y/N)** | **Blank (Y/N)** | **Time (sec)** | **Pressure** | **Pore Size (μm)** |
| --- | --- | --- | --- | --- | --- | --- |
|  |  |  |  |  |  |  |
|  |  |  |  |  |  |  |
|  |  |  |  |  |  |  |
|  |  |  |  |  |  |  |
|  |  |  |  |  |  |  |

**Site Characteristics**

| Water Quality | Sample Site | Left / u/s 1 m | Right / d/s 1 m | Instrument |
| --- | --- | --- | --- | --- |
| Air Temp (°C) |  |  |  |  |
| Water Temp (°C) |  |  |  |  |
| Conductivity (μS/cm) |  |  |  |  |
| pH |  |  |  |  |
| D.O. (ppm) |  |  |  |  |
| Secchi Disc/Tube (0.00 m) |  |  |  |  |
| Turbidity (NTU) |  |  |  |  |
| Chlorophyll *a* |  |  |  |  |
| Other: |  |  |  |  |
| Other: |  |  |  |  |

**Substrate Components** (Total = 100%) or **Dominant Substrate**:

| Type | % | Type | % | Type | % | Type | % |
| --- | --- | --- | --- | --- | --- | --- | --- |
|  |  |  |  |  |  |  |  |

#### **Lake Site Characteristics**

| MAX Site Depth (m) | | Distance from Shore Sampled (m) | Depth Sampled (m) | |
| --- | --- | --- | --- | --- |
|  | |  |  | |
| Is Lake Stratified?  (circle one) | Yes \| No Unknown | Was sample collected above Thermocline?  (circle one) | Yes \| No | Thermocline Depth (m): |

#### **Habitat Classification:**

| **Littoral** (photic zone) | | | | | **Offshore** | |
| --- | --- | --- | --- | --- | --- | --- |
| Vegetated (wetland) | Rocky substrate, limited vegetation | Silt substrate, limited vegetation | Sandy shore (e.g. beach) | Submerged woody debris, limited vegetation | Benthic | Pelagic |

#### **Biological Components** (Total = 100%) or Dominant

| Vegetation | Type 1 | % | Type 2 | % | Type 3 | % | Type 4 | % |
| --- | --- | --- | --- | --- | --- | --- | --- | --- |
| Aquatic |  |  |  |  |  |  |  |  |
| Riparian |  |  |  |  |  |  |  |  |
| Aquatic Animals |  | | | | | | | |

**Floodplain Components**

| Floodplain Use: | Bank (% slope): | Channel Cover (%): |
| --- | --- | --- |

**Weather Conditions** – At sampling Past 24 h

| 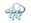 | 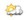 | 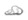 | 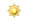 |  | 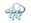 | 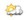 | 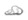 | 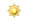 |
| --- | --- | --- | --- | --- | --- | --- | --- | --- |
| Wind Speed |  | Wind Direction |  |  | Wind Speed |  | Wind Direction |  |
| Weather Notes: | | | |  |  | | | |

**Photographs**

| Category | Photo File Nos. |
| --- | --- |
| Site |  |
| Aquatic/Terrestrial Habitats |  |
| Algae, if present |  |
| 10 cm water bottom of 5 gal bucket |  |
| Substrate bottom of 5 gal bucket |  |
| Filter after filtering |  |
| Completed field sheets |  |

Tube Label(s) Completed: Yes 🞏 No 🞏 | Field Sheet Completed: Yes 🞏 No 🞏

*Lotic waterbodies

eDNA Collection Data Sheet - GEN-FISH, University of Windsor, Windsor, ON

| Project Name |  | |
| --- | --- | --- |
| **Site Code** |  | |
| **Collector:** | | **Recorder:** |

| Date (ddmmyy) |  | | |
| --- | --- | --- | --- |
| Arrival Time | : (24h) | Departure Time | : (24h) |
| Sampling Start Time : (24h) | | Sampling Stop Time : (24h) | |

**Sample Location**

| Waterbody Name |  | | | | |
| --- | --- | --- | --- | --- | --- |
| Latitude (dd.ddddd°) |  | | Longitude  (-d.ddddd°) | |  |
| **Narrative Locality:** | | | | | |
| Paired site **(Y/N)?** | | **Upstream \| Downstream** | | Paired Site Code: | |

eDNA Sampling Method

| Gear Type | | When Filtered?  (ddmmyy; xx:xx) |
| --- | --- | --- |
| Where Filtered?  (onsite/lab/other) | | Time until samples  Frozen (hrs): |
| Filter Preservation  Method: | Other: | |

| **Sample No.** | **Volume (ml)** | **Pre-Filter (Y/N)** | **Blank (Y/N)** | **Time (sec)** | **Pressure** | **Pore Size (μm)** |
| --- | --- | --- | --- | --- | --- | --- |
|  |  |  |  |  |  |  |
|  |  |  |  |  |  |  |
|  |  |  |  |  |  |  |
|  |  |  |  |  |  |  |
|  |  |  |  |  |  |  |

**Site Characteristics**

| Water Quality | Sample Site | Left / u/s 1 m | Right / d/s 1 m | Instrument |
| --- | --- | --- | --- | --- |
| Air Temp (°C) |  |  |  |  |
| Water Temp (°C) |  |  |  |  |
| Conductivity (μS/cm) |  |  |  |  |
| pH |  |  |  |  |
| D.O. (ppm) |  |  |  |  |
| Secchi Disc/Tube (0.00 m) |  |  |  |  |
| Turbidity (NTU) |  |  |  |  |
| Chlorophyll *a* |  |  |  |  |
| Other: |  |  |  |  |
| Other: |  |  |  |  |

**Substrate Components** (Total = 100%) or **Dominant Substrate**:

| Type | % | Type | % | Type | % | Type | % |
| --- | --- | --- | --- | --- | --- | --- | --- |
|  |  |  |  |  |  |  |  |
|  |  |  |  |  |  |  |  |

#### **Stream Site Dimensions**

| Stream Width (m) | |  | | Habitat Type: Riffle \| Run \| Pool | | | |
| --- | --- | --- | --- | --- | --- | --- | --- |
| Hydraulic Head (mm) | |  | | Distance from Shore Sampled (m) | |  | |
| Depth Sampled (m) | |  | | MAX Site Depth (m) | |  | |
| Depth measurements (along horizontal transect): | | | | | | | |
| 1 **(nearshore)** | 2 | | 3 **(mid-stream)** | | 4 | | 5 **(nearshore)** |
|  |  | |  | |  | |  |

#### **Stream Flow**

| Stream Flow (Circle one) | None \| Slow \| Medium \| Fast | | Flow Meter Used | |  | |
| --- | --- | --- | --- | --- | --- | --- |
| Stream Velocity Measurements (m/s): | | | |  | | |
| Left: | | Middle: | | | | Right: |
| Flow Relative to Typical Levels: Low \| Typical \| High | | | | | | |

#### **Biological Components** (Total = 100%) or Dominant

| Vegetation | Type 1 | % | Type 2 | % | Type 3 | % | Type 4 | % |
| --- | --- | --- | --- | --- | --- | --- | --- | --- |
| Aquatic |  |  |  |  |  |  |  |  |
| Riparian |  |  |  |  |  |  |  |  |
| Aquatic Animals |  | | | | | | | |

**Floodplain Components**

| Floodplain Use: | Bank (% slope): | Channel Cover (%): |
| --- | --- | --- |

**Weather Conditions** – At sampling Past 24 h

| 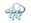 | 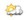 | 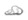 | 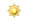 |  | 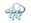 | 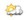 | 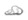 | 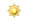 |
| --- | --- | --- | --- | --- | --- | --- | --- | --- |
| Wind Speed |  | Wind Direction |  |  | Wind Speed |  | Wind Direction |  |
| Weather Notes: | | | |  |  | | | |

**Photographs**

| Category | Photo File Nos. |
| --- | --- |
| Site |  |
| Aquatic/Terrestrial Habitats |  |
| Algae, if present |  |
| 10 cm water bottom of 5 gal bucket |  |
| Substrate bottom of 5 gal bucket |  |
| Filter after filtering |  |
| Completed field sheets |  |

Tube Label(s) Completed: Yes 🞏 No 🞏 | Field Sheet Completed: Yes 🞏 No 🞏

**GEN-FISH eDNA Sampling Protocol**

Water sampling and on-site filtration using “DIY” peristaltic pump system

**
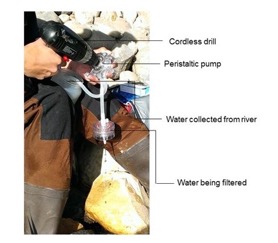
**

**Overview**: Parts to assemble this “DIY” water filtration system cost ~$600–800, plus the cordless drill (~$200) which powers it. Rate of filtration varies with turbidity, but three 0.5–1 L water samples plus a field blank can be filtered in ~20–45 minutes.

A. Materials (see Figure 1)

See ***Appendix*** *C****. Recommended Supplies List for eDNA Sampling*** for ordering details.

Quantities per site based on three biological replicates (water samples) plus one negative control (NC, field blank) per site.

- 1. **Peristaltic pump head** with key, locknuts, and bolts, stored in an individual clean bag).
  2. **Filter holder (stored in an individual clean bag).**
  3. **Silicone tubing** cut into ~75 cm long pieces, stored in an individual clean bag.
  4. **Adjustable wrench** for filtration system set-up.
  5. **Handheld screwdriver,** flathead.
  6. **Cordless drill** with flathead drill bit.
  7. **Extra drill batteries.**
  8. **Plastic sealable container** for transporting above items.
  9. **Deionized water or sealed bottled water (500 mL)**, minimum four per site
     1. Bottle #1: Rinsing tubing upon arrival at site- keep bottle for collecting water
     2. Bottle #2: Negative control filtering- keep bottle for collecting water
     3. Bottle #3: Rinsing tubing after sampling completed at site
     4. Bottle #4: Extra
  10. **Sterile metal forceps.** These are easily sterilized in the field with ethanol
      1. **Sterile 15 mL tubes filled with 70 - 95% ethanol** for dipping forceps. Minimum one per sampling day and can be reused
      2. **Lighter,** such as a barbecue lighter.
  11. If using **liquid DNA preservative (ethanol or RNAlater)**, include:

1. **Sterile screw-top tubes (skirted or non-skirted, Dnase and Rnase free)**, prefilled and prelabeled 2/3 with molecular-grade ethanol (preferably 99%, no less than 95%) or RNAlater. Four per site, plus unlabeled backups.
2. **Storage boxes** or f**alcon tube rack** to hold tubes upright.

If using **self-indicating silica as DNA preservative (liquid-free),** include:

1. **Coin envelopes** prelabeled. Four per site, plus extras.
2. **Plastic specimen bags,** sterile, prefilled with silica and use to store coin envelopes. Three bags per site (**#1** for negative control, **#2** for water samples and **#3** for keeping both bags #1 and #2 from a single site, together)
3. **Sampler spoons,** sterile, to fill specimen bags with silica, one per site. One scoop holds ~15 grams of silica
4. **Self-indicating silica,** prefilled or can fill specimen bags in field. ~150 grams per site. ~30 grams for negative control specimen bag #1, ~90-100 grams for water replicate specimen bag #2. Extra to top up as needed as silica becomes saturated.
   1. **Sharpies and ethanol-proof markers.**
   2. **ELIMINase or sodium hypochlorite solution** (concentrated bleach contains chlorine concentration of 5.25-8% or 52.5-80 grams per litre or 52,500 – 80,000 ppm; for example, one part 5% bleach to nine parts cold water will make a 10% bleach solution and the concentration of chlorine in the solution is 5000 ppm, which is ideal for high level disinfection. Bleach solutions at 10, 20 or 50 % can be made daily and stored in an opaque container away from light), ~One litre of bleach solution per site or 30 mL ELIMINase. ELIMINase is non-toxic and does not leave behind harmful residues but all ELIMINase waste and bleach must be rinsed and the wastewater stored for safe disposal. If you opt to use bleach you will need to bring enough deionized water to be able to submerge the equipment as well as a large container to store waste bleach for safe disposal after sampling.
   3. **Paper towels.**
   4. **Clean containers or large bags** to keep everything in, as well as storing waste for disposal.
   5. **Filter membranes,** Four per site, plus backups.

**
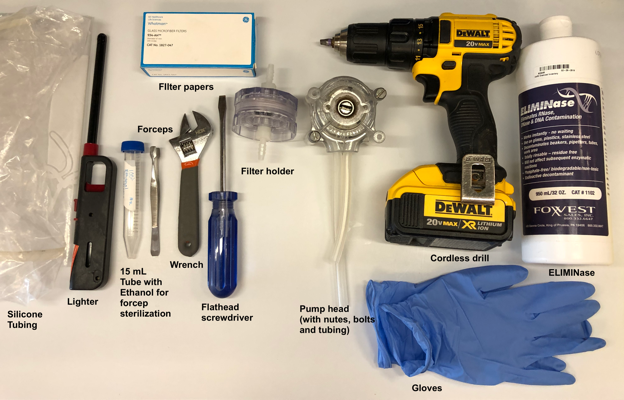
**

**Figure 1**. Image of materials needed for assembling and operating DIY peristaltic pump.

B. Clean Procedures

Given the sensitivity of the eDNA techniques, avoiding contamination of field samples is critical:

- All sampling equipment must be kept separate from other field gear (e.g., nets, clothing, truck bed).
- The peristaltic pump tubing and filter holder are also possible sources of contamination since they are exposed to the surrounding environment at every sampling site. Do not handle these (or any other non-sterile items) while wearing sterile gloves.
- Always wear fresh gloves while handling sterile items and samples.
- When in doubt, change your gloves.
- Keep contaminated, used or dirty field materials bagged and separate from clean equipment to minimize chances of contamination.
- **Always stay downstream of sampling locations.**

C. Sample Filtration

1. Having a piece of tubing that is 75 cm long will allow you to comfortably assemble and filter water. This end that pulls the water through the peristaltic pump will be called the “in-flow” end.
2. Pick up a sterile piece of tubing from the middle of the tube. Avoid picking up tubing from the ends because the one end of the tubing is going to be placed in your water sample and you don’t want to contaminate it, even if you’re wearing gloves.
3. To close the pump head, you will need to ensure that the tubing is tightly wrapped around the pump and may need to be held in place while you close the pump head. Place the nuts and bolts in the pump head and tighten using the screwdriver and wrench

*DO NOT TOUCH ANY INTERIOR PART OF THE FILTER HOLDER UNLESS IT’S WITH STERILE FORCEPS.*

1. To determine the top and bottom of the filter holder, see **Fig. 5**, or use the small white nozzle to orient. The top side of the filter holder with have a small white nozzle that can be loosened or tightened to decrease pressure build-up in the filter if the filter starts to clog.
2. The filter holder has two identical large white nozzles that are placed in the middle of the holder, on both the top and bottom. One end of the tubing can be inserted into top of the filter holder.

D. Preparation

Decontaminate bags and supplies

1. Replace or wash the bags, the pump head, filter holder, tubing, and tools. Equipment can be washed in a clean sink wearing gloves using the flow chart instructions provided in **Appendix B**
2. Hang bags to dry in a freshly sanitized area, free of contaminant DNA. Preferably in a closed indoor area away from sources of contaminants such as fish DNA, areas where dissections or PCR are conducted.
3. If you have access to a PCR workstation with UV light irradiation equipment, it can then be UV treated and bagged in the workstation.
4. Wearing gloves, the pump head and filter holder can be placed in individual clean bags. Tubing pieces can be folded in half to form a “U-shape” and placed in another bag with the inlet and outlet ends of the U-shaped tube at the bottom of the bag. This way, when you need to take a tube out, you would be handling the tube from the middle of the bend in the U. Tools can be placed in another bag.
5. All items can be packed into a larger container or tote bag with heavier items at the bottom and lighter and more fragile components at the top. Having multiple bags or containers can help simplify packing. In one bag, you can place the tubing bag at the bottom, followed by the pump bag, filter holder bag and lastly with bag of tools for assembling filter. In a smaller bag, you can store the clean filters, forceps (in a plastic bag to prevent injury), tube of ethanol or bleach to sanitize forceps and lighter.
6. For each site you visit, you will want to have a piece of tubing, plus extras.

Prepare filter storage containers

Preparing filter storage ahead of time will save time in the field and prevent contamination.

Ethanol or RNAlater

1. Label the appropriate number of tubes with site name, date and ID and other important information.
2. Prefill tubes ^2^/_3_ to ¾ full with DNA stabilizer.
3. Store upright in boxes or racks, also labeled.

Self-indicating silica

1. Label the appropriate number of coin envelopes with site name, date and ID and other important information.
2. Place three water sample coin envelopes into a plastic specimen bag, and envelop for control into a secondary plastic bag
3. Prefill specimen bag for three water samples with ~90-100 grams (seven scoop spoons) of silica
4. Prefill specimen bag for one control sample with ~30 grams (two scoop spoons) of silica
5. Store specimen bags in a secondary bag for each site.

E. Arrival at sampling site

“Housekeeping”

- Upon arrival at sampling site, determine who will be the Data Collector, DIY Sampling Assistant, and Water Sampler.
- The Data Collector will record the initial site metadata, such as starting time and any other parameters, except water chemistry.
- Meanwhile, the DIY Sampling Assistant and Water Sampler will start to assemble the filtration device.

Assembling DIY Filtration

- - - 1. Wearing sterile gloves, use the cordless drill with a flat head drill bit or the hand-held flat-head screwdriver to open the pump head. This may require the help of another person who can hold the nuts in place while the screw is undone, eliminating the need to place the filtration apparatus down. If the filtration system needs to be placed down, do so on clean paper towels.
      2. Place a piece of sterile tubing in the pump. To avoid any risk of contamination, leave enough room on one end of the tubing to allow it to reach the bottom of your water container; this will be the “inflow” end; the other end will be the “outflow.”
      3. Ensure that the tubing is tightly wrapped around the pump and hold it in place while you close the pump head.
      4. Replace the nuts and screw on the pump head, tightening using the wrench and screwdriver.
      5. Find the top side of the filter holder by looking for the small white plastic screw. The white screw should always remain *almost* screwed shut into the nozzle. If it’s too loose, water will pour out from the top and not be filtered. Insert the outflow end of the tubing onto the nozzle on the top of the filter holder.

Flush the tubing

1. Take a sealed, 500 mL bottle of water or sterile, deionized, water (**Bottle #1**), and carefully place the inflow end of the tubing into the bottle of water until it reaches the bottom of the bottle. Ensure that you do not touch any portion of the tubing that will go into the water, even with gloved hands.
2. Place the appropriate flat head drill bit into the drill and insert drill head into pump head.
3. Set the gear selector switch on the drill to low gear or “1,” and press on the speed trigger. Water will start to be drawn from the water bottle, and into the tubing and out through the outflow filter holder. If water does not begin to filter through, and you’re seeing bubbles forming in the water bottle, you need to reverse the switch to change the direction of the drilling by using the forward/reverse button. You can do this by pushing on the “REVERSE SWITCH” button on the drill (see **Fig. 2**, labelled drill image)
4. Increase the gear setting to the higher setting, “2,” and continue to filter the water through the tube at a faster rate. Flushing the tubing with water at a fast speed with help to draw out any debris or contaminants.

Insert the filter into the filter holder

While one person holds the pump head and ensures the tubing inflow and outflow ends do not touch anything, the other will place a clean filter onto the filter holder. Do not touch any interior part of the filter holder unless it is with sterile forceps.

1. Unscrew the filter holder while holding the two parts vertically. Inside, there will be an orange coloured O-ring that rests on the top part of the filter holder and holds the filter in place during filtration. The bottom of the filter holder has a black surface that the filter rests on. Holding the top and bottom of the filter holder vertically will prevent the O-ring from falling on the ground and getting contaminated (as labelled in **Fig. 8**)
2. If using metal forceps, dip them into your 15 mL tube of ethanol, holding the pincers of the forceps facing down and flame them with your lighter to ensure sterility. It is crucial that the forceps dipped in ethanol are held in a manner that allows for the ethanol to drip down and not upright, which can lead to burns.
3. Use clean gloves to open the box of filters without touching the inside of the box. Using sterile forceps, pick up a filter and place it on the black filter area inside the holder.
4. Close the filter holder while holding the two parts vertically.
5. Using your sterile forceps, close the box of filters.

*You’re now ready to start filtering water samples.*

Take a field blank or negative control

From this point, the Water Sampler who will collect the water samples with gloves must not come into contact with any filtration equipment until gloves are removed.

1. While one person holds the pump head and watches to ensure the tubing inflow and outflow end do not touch anything, the second person can use a sterile pair of forceps to place a clean filter onto the filter holder
2. Take a sealed, 500 mL bottle of water or sterile, deionized water (**Bottle #2**), and carefully place the inflow end of the tubing into the bottle of water, ensuring that you do not touch any portion of the tubing that will go into the bottle.
3. Change the gear setting on the drill back to low or “1” and keep this setting for the remainder of filtering water samples.
4. Press on the speed trigger and filter the water. Keep an eye on the tubing as it may become suctioned to the side of the bottle. If it becomes suctioned, move the water bottle down and away from the tubing to detach it.
5. Once the there is no water left in the bottle, continue to gently press the trigger to draw out any excess water from the filter.
6. One person can hold the pump head while the other carefully opens the filter holder.
7. Transfer the filter to the filter storage using sterile, disposable forceps or after sterilizing forceps. **Allow nothing to come in contact with the inside of the tube except for filter and sterile forceps**:
8. Ethanol or RNAlater storage Liquid preservative storage: fold the filter in half on the side that contains the DNA, so the filtered material is enclosed, then fold in half two more times. Place the folded filter paper into preservative tube, being careful to not touch the inner portion of the tube or lid without your forceps. Screw the cap tightly onto the vial and place into the storage box.
9. Self-indicating silica preservative storage: Do not fold this filter. Place the filter into the coin envelope and into a plastic specimen bag with silica. The bag can be pre-filled with ~30 grams of silica or add silica now. Seal the plastic specimen bag and set aside.
10. Using sterile forceps or after sterilizing forceps, place a new filter into the filter holder.

Collect water samples from the site

The Water Sampler will collect the appropriate number of water samples from the site referring to your specific sampling design. To decrease the number of bottles needed, you can use the same bottle for each of the three bio-replicates. At this point you should have two empty water bottles that can be used to collect water (Bottles #1 and 2).

In most cases you will be collecting water from the water surface, without stepping into the water. If you must step into the water, ensure you are downstream from your sampling site. from an access point such as a bank

Filter water samples from the site

1. Keeping the drill gear setting on low or “1,” press on the trigger and filter the water.

- - - - - Keep an eye on the tubing, as it may become suctioned to the side of the bottle.
        - Filter the water as you’ve filtered the blank or negative control and depending on the level of turbidity, you may need to slow down if you see water is being backed up in the top portion of the filter holder.
        - You can adjust the pressure in the filter by gently releasing the small white nozzle at the top of the filter.
        - Filter as much water as you can until the filter cannot filter any additional volumes
      1. Record the volume of water filtered using a graduated cylinder.
      2. Remove the inflow end of the tubing from the water sample and continue to press on the trigger to without any water in the inflow to draw out excess water and moisture.
      3. Transfer the filter using sterile, disposable forceps or after sterilizing forceps to:

1. Ethanol or RNAlater storage: fold the filter in half so the filtered material is enclosed, then fold in half twice more. Place the folder filter paper into preservative tube, being careful to not touch the inner portion of the tube or lid with your forceps.
2. Self-indicating silica preservative storage: Do not fold this filter. Place the filter into the coin envelope and into a plastic specimen bag with silica. The bag can be pre-filled with ~90-100 grams of silica or add silica now. Seal the plastic specimen bag and set aside.
   - - 1. Using sterile forceps or after sterilizing forceps, place a new filter into the filter holder.
       2. Repeat this procedure for each water sample from the site and preserve filters accordingly:
3. Liquid preservative storage: fold the filter in half on the side that contains the filtered material, and in half two more times. Place the folded filter paper into preservative tube being careful to not touch the inner portion of the tube or lid without forceps.
4. Self-indicating silica preservative storage: Do not fold this filter. Place the filter into the coin envelope and into the **same plastic specimen bag with silica as previously filtered sample**. The bag is pre-filled with ~90-100 grams of silica, and more may need to be or add silica now. Seal the plastic specimen bag and set aside. Plastic specimen bag with blank and plastic specimen bag with field water samples can be placed into a third specimen bag to keep samples from a single site organized.

Once sampling at that site is complete

Have three bags ready:

1. **Dirty Equipment Bag**
   - For materials that will be cleaned once back in the lab
   - Filter holder
   - Pump head
   - Silicone tubing
2. **Clean Bag**
   - For materials that will remain in a clean area
   - Binders and data sheets
3. **Disposal Bag** (cleaned once back in the lab):
   - For materials that will be stored away from clean equipment and will be disposed of upon return to lab or field station
   - gloves, disposable forceps, contaminated filters, empty water bottles
4. To disassemble the pump, which will be done after sampling at each and every site, place a sealed water bottle (Bottle #3) into the inflow end and flush water through the empty filter holder by switching the gear speed to “2,” until the water bottle (and tubing) is empty.
5. Remove the filter holder from the tubing by holding the out-flow end of the tube in one hand and filter holder in the other.
6. Place the filter holder in a designated bag that remains in the Clean Bag.
7. Unscrew the bolts screw and nuts on the pump head using the screwdriver and wrench.
8. Gently pull apart the top and bottom portion of the pump head. This may be a bit difficult due to the suction created between the silicone tubing.
9. Remove the tube and place in the Dirty Equipment Bag, dedicated to dirty tubes that will not be reused until cleaned again.
10. Put the pump head together again, loosely, and place pump head in the Clean Bag that remains in a clean area.
11. Discard gloves into a bag that is stored away from clean equipment and will be disposed of upon return to lab or field station .
12. Filters should be stored in a cool, dry and dark area until transported back to back and stored accordingly upon return to lab, field-station or house.
13. Collect any additional metadata and sampling data.
14. Repeat process at next site.

**Label ID for samples**

Minimum requirements for sample labels for each 2 mL tube, 15 mL tube or sample container:

- the site name or abbreviation for site
- sub-sample information to distinguish between a blank or negative control and eDNA water sample
- the replicate number for eDNA water samples and date sampled

Any additional information if needed can be given on the label as needed and more details can be provided on metadata sheets

Notes and Precautions

- If you’re going to be doing any other sampling in the water, please make sure that you take the eDNA samples first, followed by any other sampling in the water body. If you’re going to be taking any water chemistry parameters, this should take place last so you’re not carrying over contaminants from one site to another because cleaning sensitive water parameter equipment may not be possible in field.
- If any part from the pump or filter holder (e.g., O-ring) becomes contaminated (e.g., fell to the ground, was touched with contaminated gloves, etc.) the O-ring in the filter holder falls down or any other part from the pump or filter holder falls down, it is now contaminated and will need to be cleaned in the field.
  - With ELIMINase: To clean your equipment in the field, you can dispense a small amount of ELIMINase onto clean paper towel and wipe down and then rinse with bottled or deionized water.
  - If ELIMINase is not available, using a 10 to 20% bleach solution: submerge the contaminated part for the allotted time, and then rinse with bottled or deionized water. Perform a triple rinse with water to be sure there are not residues of bleach in the materials. Any residual bleach will degrade DNA in the sample. All waste bleach solutions must be transported back to lab or field solution for proper disposal.
    - 10% solution: 30 minutes
    - 20% solution: 15 minutes
    - 50% solution: 2 minutes
  - The O-ring can be submerged in the solution for 15-30 minutes respectively, and rinsed with plenty of water before replacement. Alternatively, a 50% bleach solution can be made and requires a contact time of 1-2 minutes- but will require a large amount of bleach. All waste bleach solutions must be transported back to lab or field solution for proper disposal
- On a windy day, you may have difficulty placing the filter on the filter holder, simply put any fallen filter papers in the Disposal Bag.
- Another challenge of a windy day is lighting the forceps for sterilizing. You can use a cardboard box to shield from the wind
- When sterilizing metal forceps, dip the forceps in ethanol, remove from ethanol tube and keep holding the forceps so ethanol does not run down to the portion you’re holding. Instead, any access ethanol would drip off of the forceps. If you dip the forceps in ethanol, flip the forceps right side up and flame, you’re risking the chance of the area you’re holding, or your hands being exposed to high heat or even burned.
- Alternative forceps sanitizing methods:
- Forceps can also be sanitized between samples by dipping in a 50% bleach solution, rinsed with deionized water and wiped dry OR
- sprayed with ELIMINase and wiped dry.

**Storage of Filters (Lab)**

- Ethanol- or RNAlater-preserved filters: Filters that are preserved in ethanol can be stored at room temperature, but we suggest storing filters at -20 to -30°C.
- Silica preserved filter: Filters that are preserved in silica can be stored at room temperature in a cool, dry location and away from light. The suggested location would be a cabinet or cupboard in a dry room.

Appendix A. Images of parts of DIY peristaltic pump, assembly and removing filter from holder to store in ethanol and silica.


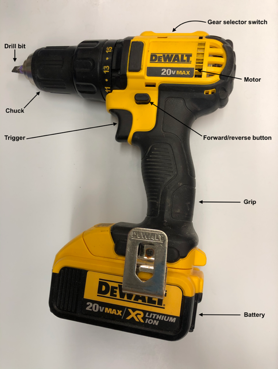


**Figure 2**. Components of cordless drill, labelled


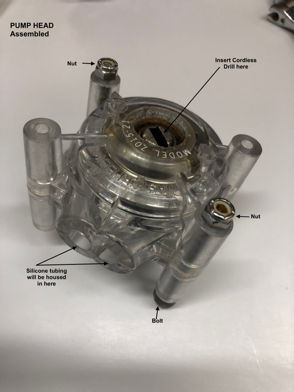


**Figure 3.** Pump head, assembled and labelled parts.


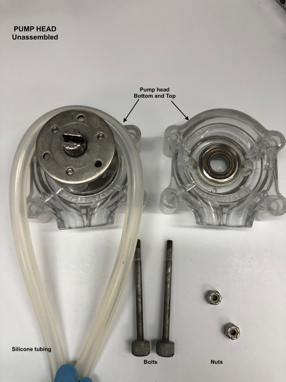


**Figure 4.** Pump head unassembled and labelled parts.


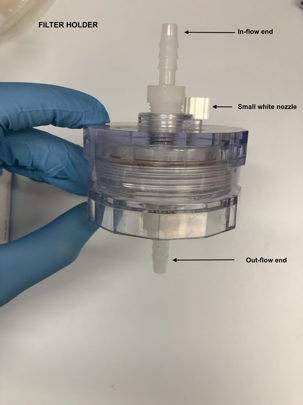


**Figure 5.** Filter holder assembled and labelled parts.


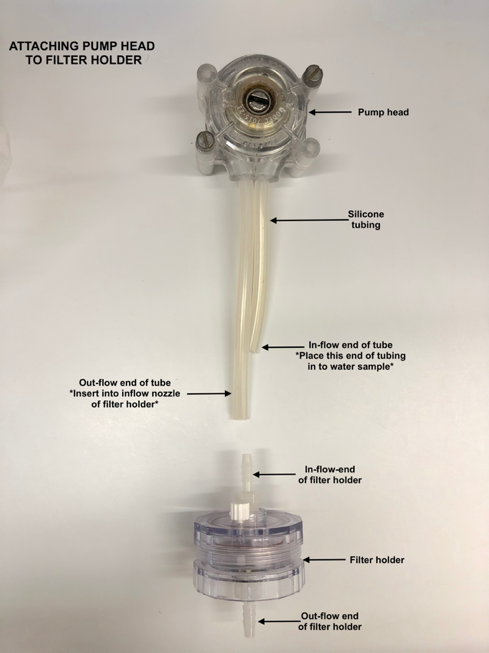


**Figure 6**. Pump head and filter holder assembled, and parts labelled to indicate site of attachment to assemble peristaltic pump with in-line filter holder.


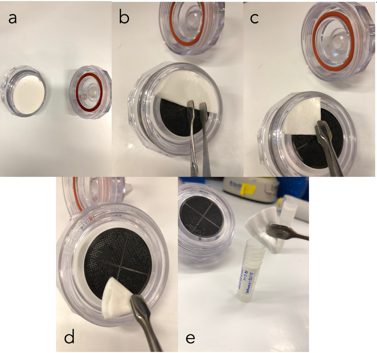


**Figure 7.** Steps labelled from a to e showing process of filter folding to preserve in liquid preservative. a: open filter holder vertically; b: fold filter with sterile forceps, inwards, onto filtrant; c and d: fold in half twice more; e: place folded filter into tube.


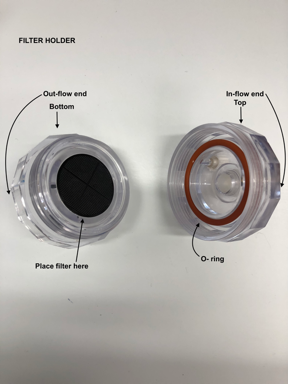


**Figure 8**. Filter holder labelled to indicate site of filter placement.

**
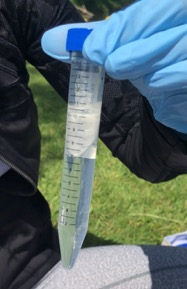

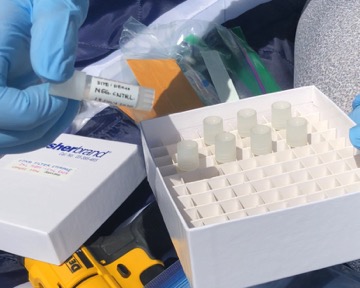
**

**b**

**a**

**Figure 9.** Storing filter in liquid preservative in either a) 15 ml falcon tube or b) 2 mL screw-top tube, both pre-filled with liquid preservative.


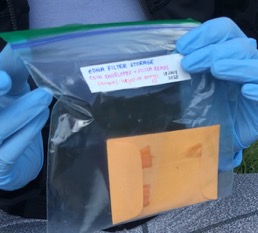

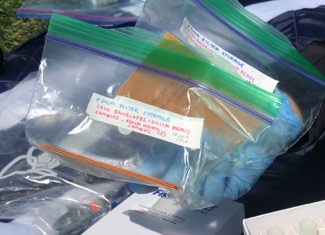


**a**

**b**

Figure **10**. Storing filter in self-indicating silica in coin envelopes. Blank or negative control filter can be placed in a) pre-labeled coin envelope, in a plastic specimen bag and water control samples can be stored in b) three individually labeled coin envelopes, in a plastic specimen bag. Bags are not pre-filled with silica, but would be filled now with ~30 grams and ~100 grams of self-indicating silica, respectively for bag in a and b.

Appendix B. Flow chart of equipment sanitation before and after field sampling

**Figure 11**. Flow chart of equipment and material sanitation procedure at Docker lab (University of Manitoba). Recyclable plastic bottles are used for assays and are not re-used. Bleach solutions that can be used to clean equipment range from 10 to 50%, with minimum contact times of 15 minutes to two minutes, respectively. Equipment is cleaned with multiple chemical sanitation steps to ensure elimination of any possible DNA contaminants. If available, equipment can be UV irradiated.

Appendix C. Recommended Supplies List for eDNA Sampling

| Item | Description | Vendor and Item Number(s) |
| --- | --- | --- |
| Peristaltic pump head | Masterflex L/S® Standard Pump Head for High-Performance Precision Tubing L/S® 15, Polycarbonate Housing, CRS Rotor | Cole Parmer: HV-07015-20 |
| Filter holder | Polycarbonate In-Line Filter Holder, 47 mm, Pall Laboratory | VWR: CA28144-257 |
| Silicone tubing, sterile | Masterflex L/S® High-Performance Precision Pump Tubing, Platinum-Cured Silicone, L/S 15; 25 ft, 4.8 mm ID | Cole Parmer: HV-96410-15 |
| Glass fibre filters | Cytiva Whatman™ 1.5 μm Binder-Free Glass Microfiber Filters, Grade 934-AH, 47 mm Circles | Fisher Scientific: 09-873DD |
| Forceps | Fisherbrand™ Filter/Membrane Stainless Steel Forceps | Fisher Scientific: 09-753-50 |
| Single-use nitrile gloves | Fisherbrand™ Powder-Free Nitrile Exam Gloves | Fisher Scientific: 191301597 (variety of sizes available) |
| Graduated container or cylinder | Fisherbrand™ Polypropylene Graduated Cylinders | Fisher Scientific: 03-007-44 |
| 1-L Nalgene water collection bottles | Fisherbrand™ Leakproof HDPE Wide-Mouth Bottles (1 litre bottles) | Fisher Scientific: 02-896-2F |
| Ethanol-proof markers | Fisherbrand™ Fine Tip Marking Pens | Fisher Scientific: 13-379-6 |
| ELIMINase | Decon™ ELIMINase™ Decontaminant | Fisher Scientific: 04-355-32 |
| Adjustable wrench | Use what you have available on hand that is comparable | Suggested Vendor: Home Depot  Crescent 6 Inch Cushion Grip Chrome Adjustable Wrench  Item: AC26CVS |
| Handheld screwdriver (flathead) | Use what you have available on hand that is comparable | Suggested Vendor: Home Depot  STANLEY Push-N-Pick Screwdriver  Item: 69-193P |
| Cordless drill | Use what you have available on hand that is comparable | Suggested: DEWALT 20V MAX Li-Ion Cordless Brushless Compact 1/2-inch Drill Driver w/ (2) Batteries 1.3Ah, Charger and Tool Bag |
| Extra cordless drill batteries | Use what you have available on hand that is comparable | Suggested: DEWALT DCB207 1.3 Ah 20V Li-Ion Compact Battery |
| ***FILTER PRESERVATION MATERIAL IN LIQUID PRESERVATIVE*** | | |
| Storage box, size appropriate to tube size. | Fisherbrand™ Cryo/Freezer Boxes (100- tube capacity) | Fisher Scientific: 03-395-465 |
| For filter storage:  Sterile tubes (2 – 15 mL) | United Scientific Supplies 15 mL Centrifuge Tubes  National Scientific™ BioStor™ 2 mL Screw Cap Vials, Skirted | Fisher Scientific (15 mL): S99410  Fisher Scientific (2 mL): 11-844-18 |
| RNAlater RNA stabilization solution | Invitrogen™ RNA*later*™ Stabilization Solution | Fisher Scientific (500 mL): [**AM7021**](https://www.fishersci.ca/shop/products/ambion-rna-i-later-i-stabilization-solution-7/am7021?keyword=true) |
| Ethanol | Ethyl Alcohol Denatured, MilliporeSigma™ | Fisher Scientific (4 L): MEX02803 |
|  |  |  |
| ***FILTER PRESERVATION MATERIALS IN SELF-INDICATING SILICA*** (no liquid preservation) | | |
| Coin envelopes, sterile | Coin Envelopes with Gummed Flaps, 2-1/4" x 3-1/2" | Suggested Vendor: Staples  Item: 438346 Model: 530164 |
| Self-indicating silica beads, sterile  *NEW addition* | 55 lb Bag Orange Indicating Silica Gel | Suggested Vendor: IMPAK  Item[: 640SGO55](https://www.impakcorporation.com/desiccants/bulk_desiccant/640SGO55) |
| Self-indicating silica beads, sterile | Silica Gel, Honeywell Fluka (metal free)  2.5 kg | Fisher Scientific: 6002003 |
| Plastic specimen bags, sterile | Minigrip™ Reclosable White Specimen Bags | Fisher Scientific: 22-310-032 |
| Sterile sampler spoons | Bel-Art™ Sterileware™ Sterile Styrene Sampler Spoons (~15 mL capacity) | Fisher Scientific: 03-990-232 |

## Fish health assessment: Sampling protocols and Figures

### Fish health assessment (gill, liver and muscle tissue samples)

Tissue Sampling Protocol for Fish Health work:

1) Please thoroughly clean dissecting tools when moving between sampling locations.

2) Please take the tissue samples as quickly as possible of freshly killed fish because the tissue will degrade after the fish is dead.

3) Use forceps to move tissue samples into 1.5ml tube already containing ~1 ml of RNA*later*.

- Important: We don’t need much tissue for this work, therefore the tissue needs to be small enough to be properly preserved by the RNA*later*. Less is more for this work!

- Roughly: - Gill samples should be around the size of a thumbnail.

- Liver samples should be a cube around the size of the tip of your baby finger.

- Muscle samples should be around the size of the tip of your thumb.

4) In between samples, wipe down tools before taking the next sample. Please continually use new Kim wipes throughout the process. You really can’t use too many!

5) After the samples have been collected, they need to be kept at room temperature or 4°C in RNA*later* for at least 24 hours to allow the solution to penetrate and preserve the tissue. If they can be kept cool until they are returned to the lab that would be ideal. Then the samples can be moved into a regular freezer (-20°C) to be stored until they can be shipped to Manitoba.

| **GEN-FISH Pilot Project to Test Utility of GEN-FISH Environmental DNA (eDNA) and Fish Health Toolkits for Community Based Monitoring of Culturally Significant Fish Species in Wood Buffalo National Park**  *Template to use for collecting fish tissue metadata*  *All fields in orange to be collected while in field, others can be filled before or after sampling* | | | | | | | | | | | | | | | | |
| --- | --- | --- | --- | --- | --- | --- | --- | --- | --- | --- | --- | --- | --- | --- | --- | --- |
| **Sample #** | **Species name** | **Common name** | **Province** | **Water body** | **Location** | **Lat** | **Long** | **Sampling Date** | **Tissue type (fin, gill, liver, muscle, etc)** | **Preserv. medium** | **Length (cm)** | **Weight (g)** | **Sex** | **Life Stage** | **Parasites present in or on the fish? (YES/ NO; if yes specify location of parasites: (IN/ ON or BOTH)** | **Quality of flesh (normal, watery, other, specify other)** |
| *Example* | *Entosphenus macrostomata* | *Vancouver lamprey* | *BC* | *Cowichan Lake* |  | *48.89* | *-124.31944* | *14-May-21* | *Fin* | *100% ethanol* | *14.3 (Larvae)* | *4.17 (Larvae)* | *-* | *larvae* | *Yes, Inside and outside* | *Normal* |
|  |  |  |  |  |  |  |  |  |  |  |  |  |  |  |  |  |
|  |  |  |  |  |  |  |  |  |  |  |  |  |  |  |  |  |
|  |  |  |  |  |  |  |  |  |  |  |  |  |  |  |  |  |
|  |  |  |  |  |  |  |  |  |  |  |  |  |  |  |  |  |
