# Supplementary material for: Community-based eDNA metabarcoding for monitoring fish biodiversity and food webs in the Peace-Athabasca Delta
Source: PeerJ. 2026 May 22;14:e21341. doi: 10.7717/peerj.21341 (PMC13200620; doi:10.7717/peerj.21341)
Supplement: Supplemental Information 2 [file peerj-14-21341-s002.docx]

Supplementary Table 1. DNA quantification measurements conducted for the eDNA extracts obtained from each sample and field negative controls (NC).

| **Site Code** | **Sample ID** | **DNA quantification** | | | | |
| --- | --- | --- | --- | --- | --- | --- |
|  | | **1** | **2** | **3** | **Average concentration** | **SD** |
| Big Egg Lake | BEL-1 | 17.6 | 17.9 | 17.9 | 17.8 | 0.173 |
| Big Egg Lake | BEL-NC | 0.119 | 0.211 | 0.217 | 0.182 | 0.055 |
| Embarras | E_1 | 13 | 13.3 | 13.1 | 13.133 | 0.153 |
| Embarras | E_NC | 0.034 | 0.0347 | 0.0347 | 0.034 | 0.0004 |
| Fletcher site A | F_S_A_1 | 4.63 | 4.38 | 4.56 | 4.523 | 0.129 |
| Fletcher site A | F_S_A_NC | too low | too low | too low | too low |  |
| Flett Creek A | FC_A_1 | 1.83 | 1.73 | 1.69 | 1.75 | 0.072 |
| Flett Creek A | FC_A_NC | too low | too low | too low | too low |  |
| Flett Creek B | FC_B_1 | 4.35 | 4.3 | 4.31 | 4.32 | 0.026 |
| Flett Creek B | FC_B_NC | too low | too low | too low | too low |  |
| Jackfish site A | J_S_A_1 | 18.8 | 20.1 | 20.1 | 19.667 | 0.751 |
| Jackfish site A | J_S_A_NC | too low | too low | too low | too low |  |
| Jackfish site B | JS_B_1 | 9.93 | 10.3 | 10.4 | 10.21 | 0.2483 |
| Jackfish site B | JS_B_NC | 0.217 | 0.205 | 0.185 | 0.202 | 0.016 |
| Old Fort | OF_1 | 10.4 | 10.7 | 10.7 | 10.6 | 0.173 |
| Old Fort | OF_NC | too low | too low | too low | too low |  |
| Old Fort Site B | OF_S_B_1 | 11.3 | 11.3 | 11.4 | 11.333 | 0.058 |
| Old Fort Site B | OF_S_B_NC | too low | too low | too low | too low |  |
| Pelican Creek | P_CR_1 | 8 | 7.93 | 8.2 | 8.043 | 0.140 |
| Pelican Creek | P_CR_NC | too low | too low | too low | too low |  |
| Prairie River @ Parks Cabin | PC_1 | 24 | 27.1 | 26.3 | 25.8 | 1.609 |
| Prairie River @ Parks Cabin | PC_NC | too low | too low | too low | too low |  |
| Peace River A | PR_A_1 | 6.3 | 6.25 | 6.21 | 6.253 | 0.045 |
| Peace River A | PR_A_NC | too low | too low | too low | too low |  |
| Peace River B | PR_B_1 | 6.01 | 5.92 | 6.09 | 6.007 | 0.085 |
| Peace River B | PR_B_NC | too low | too low | too low | too low |  |
| Quatre Fourches | QS_1 | 11.4 | 11.4 | 11.8 | 11.533 | 0.231 |
| Quatre Fourches | QS_NC | too low | too low | too low | too low |  |
| Rapids A | R_A_1 | 3.21 | 3.23 | 3.12 | 3.187 | 0.0586 |
| Rapids A | R_A_NC | too low | too low | too low | too low |  |
