# Supplementary material for: Community-based eDNA metabarcoding for monitoring fish biodiversity and food webs in the Peace-Athabasca Delta
Source: PeerJ. 2026 May 22;14:e21341. doi: 10.7717/peerj.21341 (PMC13200620; doi:10.7717/peerj.21341)
Supplement: Supplemental Information 3 [file peerj-14-21341-s003.docx]

Supplementary Table 2. Species list per collection site generated from eDNA detection in the sampled water bodies using the MetaWorks pipeline (12S half and whole filter analysis) and VertCOI. Matches in fish detection based on 12S whole filter and the COI (VertCOI) molecular marker have been indicated (X). The total number of reads and statistical support obtained from the MetaWorks pipeline (12S half filter) have been added for each species classification. If the species of interest was not present with the 12S half filter, the number of reads represents the marker with the species present. Reads generated per taxon across technical replicates were averaged (Average reads). The full number of raw reads obtained per sample are shown in Supplementary Table 3.

| **Site Code** | **Sample name** | **Species** | **12S** | **12S (whole filter)** | **VertCOI** | **12S half bootstrap** | **12S whole bootstrap** | **COI bootstrap** | **Average reads** |
| --- | --- | --- | --- | --- | --- | --- | --- | --- | --- |
|  |  |  | **(half filter)** |  |  |  |  |  |  |
| Embarras | E_1_I, III | *Lota lota* | X | X | - | 1 | 1 | - | 2,399 |
| (N=10) | E_1_I, II, III | *Esox lucius* | X | X | X | 0.99 | 0.99 | 1 | 25,680 |
|  | E_1_I, II, III | *Notropis atherinoides* | X | X | - | 1 | 0.98 | - | 12,249 |
|  | E_1_I, II | *Sander vitreus* | X | X | X | 1 | 1 | 1 | 1,128 |
|  | E_1_I, II, III | *Notropis hudsonius* | X | X | X | 0.99 | 0.99 | 1 | 677 |
|  | E_1_I, II, III | *Hiodon alosoides* | X | X | X | 1 | 1 | 1 | 15 |
|  | E_1_II, III | *Perca flavescens* | X | - | - | 1 | - | - | 7 |
|  | E_1_I, II, III | *Catostomus commersonii* | X | X | X | 0.73 | 0.75 | 1 | 18,340 |
|  | E_1_II | *Percopsis omiscomaycus* | - | - | X | - | - | 1 | 200 |
|  | E_1_II | *Rhinichthys cataractae* | X | - | - | 0.91 | - | - | 3 |
|  |  |  |  |  |  |  |  |  |  |
| FletcherA | F_S_A_1_I, II, III | *Lota lota* | X | X | - | 1 | 1 | - | 475 |
| (N=9) | F_S_A_1_I, II, III | *Esox lucius* | X | X | X | 0.99 | 0.99 | 1 | 6,884 |
|  | F_S_A_1_I, II, III | *Notropis atherinoides* | X | - | - | 1 | - | - | 133 |
|  | F_S_A_1_I, II, III | *Sander vitreus* | X | - | - | 1 | - | - | 2,517 |
|  | F_S_A_1_I, II, III | *Hiodon alosoides* | X | X | X | 1 | 1 | 1 | 13,542 |
|  | F_S_A_1_II | *Catostomus catostomus* | X | X | - | 0.99 | 0.99 | - | 847 |
|  | F_S_A_1_III | *Perca flavescens* | X | - | - | 1 | - | - | 4 |
|  | F_S_A_1_I, II, III | *Catostomus commersonii* | X | X | X | 0.73 | 0.75 | 1 | 4,901 |
|  | F_S_A_1 | *Oncorhynchus spp.* | - | X | - | - | 0.97 | - | 22,659 |
|  |  |  |  |  |  |  |  |  |  |
| FlettCreekA | FC_A_1_I, II, III | *Esox lucius* | X | X | X | 0.99 | 0.99 | 1 | 26,731 |
| (N=10) | FC_A_1_I, II, III | *Notropis atherinoides* | X | X | - | 1 | 0.98 | - | 23,635 |
|  | FC_A_1_I, II | *Sander vitreus* | X | - | X | 1 | - | 1 | 2,010 |
|  | FC_A_1_I, II, III | *Hiodon alosoides* | X | - | X | 1 | - | 1 | 6 |
|  | FC_A_1_II | *Perca flavescens* | X | X | - | 1 | 1 | - | 64 |
|  | FC_A_1_III | *Lota lota* | X | X | - | 1 | 1 | - | 3,551 |
|  | FC_A_1_I, III | *Catostomus commersonii* | X | - | X | 0.73 | - | 1 | 2,186 |
|  | FC_A_1_II, III | *Percopsis omiscomaycus* | - | - | X | - | - | 1 | 113 |
|  | FC_A_1_III | *Notropis hudsonius* | X | X | X | 0.93 | 0.93 | 1 | 5,977 |
|  | FC_A_1_I, III | *Coregonus spp.* | X | - | X | 0.92 | - | 0.04 | 2,475 |
|  |  |  |  |  |  |  |  |  |  |
| FlettCreekB | FC_B_1_I, II, III | *Catostomus catostomus* | X | X | - | 0.99 | 0.99 | - | 4,472 |
| (N=11) | FC_B_1_I, II, III | *Lota lota* | X | - | - | 1 | - | - | 454 |
|  | FC_B_1_I, II, III | *Esox lucius* | X | X | X | 0.99 | 0.99 | 1 | 122,788 |
|  | FC_B_1_I, II, III | *Notropis atherinoides* | X | X | X | 1 | 0.98 | 0.988 | 101,356 |
|  | FC_B_1_I, II | *Sander vitreus* | X | X | X | 1 | 1 | 1 | 896 |
|  | FC_B_1_I, II, III | *Perca flavescens* | X | X | X | 1 | 1 | 1 | 36,964 |
|  | FC_B_1_I, II, III | *Hiodon alosoides* | X | X | X | 1 | 1 | 1 | 17,722 |
|  | FC_B_1_II | *Notropis hudsonius* | X | X | - | 0.99 | 0.99 | - | 1,643 |
|  | FC_B_1_I, II | *Percopsis omiscomaycus* | - | - | X | - | - | 1 | 680 |
|  | FC_B_1_II, III | *Catostomus commersonii* | X | - | X | 0.73 | - | 1 | 6,578 |
|  | FC_B_1_I, II, III | *Coregonus spp.* | X | - | - | 0.92 | - | - | 2,412 |
|  |  |  |  |  |  |  |  |  |  |
| JackfishA | J_S_A_1_I, II, III | *Lota lota* | X | X | - | 1 | 1 | - | 5,825 |
| (N=12) | J_S_A_1_I, II, III | *Esox lucius* | X | X | X | 0.99 | 0.99 | 1 | 120,487 |
|  | J_S_A_1_I, II, III | *Notropis atherinoides* | X | X | - | 1 | 0.98 | - | 49,223 |
|  | J_S_A_1_I, II, III | *Sander vitreus* | X | X | X | 1 | 1 | 1 | 14,705 |
|  | J_S_A_1_I, II, III | *Perca flavescens* | X | - | - | 1 | - | - | 6,963 |
|  | J_S_A_1_I, II | *Notropis hudsonius* | X | X | - | 0.99 | 0.93 | - | 722 |
|  | J_S_A_1_I, II, III | *Hiodon alosoides* | X | X | X | 1 | 1 | 1 | 12,449 |
|  | J_S_A_1_III | *Couesius plumbeus* | - | X | - | - | 0.99 | - | 4,076 |
|  | J_S_A_1_I, III | *Catostomus commersonii* | X | - | X | 0.73 | - | 1 | 875 |
|  | J_S_A_1_I | *Rhinichthys cataractae* | X | X | - | 0.91 | 0.9 | - | 6,050 |
|  | J_S_A_1_I, II, III | *Oncorhynchus spp.* | X | X | - | 0.07 | 0.07 | - | 50 |
|  | J_S_A_I, 3 | *Coregonus spp.* | - | X | - | - | 0.95 | - | 5,988 |
|  |  |  |  |  |  |  |  |  |  |
| JackfishB | JS_B_1_I | *Catostomus catostomus* | X | - | - | 0.99 | - | - | 1,400 |
| (N=12) | JS_B_1_I, II, III | *Lota lota* | X | X | - | 1 | 1 | - | 23,895 |
|  | JS_B_1_I, II, III | *Esox lucius* | X | X | X | 0.99 | 0.99 | 1 | 108,793 |
|  | JS_B_1_I, II, III | *Notropis atherinoides* | X | X | - | 1 | 0.98 | - | 5,047 |
|  | JS_B_1_I, II, III | *Sander vitreus* | X | X | X | 1 | 1 | 1 | 11,542 |
|  | JS_B_1_I, III | *Perca flavescens* | X | - | - | 1 | - | - | 4 |
|  | JS_B_1_I, II, III | *Hiodon alosoides* | X | X | X | 1 | 1 | 1 | 2,597 |
|  | JS_B_1_II | *Prosopium williamsoni* | X | - | - | 0.97 | - | - | 1,464 |
|  | JS_B_1_I, III | *Catostomus commersonii* | X | X | X | 0.73 | 0.75 | 1 | 1,097 |
|  | JS_B_1_I, II, III | *Rhinichthys cataractae* | X | X | X | 0.9 | 0.9 | 0.95 | 1,503 |
|  | JS_B_1_I, II, III | *Oncorhynchus spp.* | X | X | - | 0.07 | 0.07 | - | 222 |
|  | JS_B_1_I | *Coregonus spp.* | X | X | - | 0.92 | 0.95 | - | 557 |
|  |  |  |  |  |  |  |  |  |  |
| OldFortB | OF_S_B_1_I, II | *Catostomus catostomus* | X | X | - | 0.99 | 0.99 | - | 1,721 |
| (N=11) | OF_S_B_1_I, II, III | *Lota lota* | X | - | - | 1 | - | - | 4,334 |
|  | OF_S_B_1_I, II, III | *Esox lucius* | X | X | X | 0.99 | 0.99 | 1 | 29,958 |
|  | OF_S_B_1_I, II, III | *Notropis atherinoides* | X | X | - | 1 | 0.98 | - | 27,325 |
|  | OF_S_B_1_I, II, III | *Sander vitreus* | X | X | X | 1 | 1 | 0.996 | 5,322 |
|  | OF_S_B_1_I, II, III | *Perca flavescens* | X | - | - | 1 | - | - | 8 |
|  | OF_S_B_1_I, II, III | *Hiodon alosoides* | X | X | X | 1 | 1 | 1 | 12,607 |
|  | OF_S_B_1_I,II, III | *Catostomus commersonii* | X | X | X | 0.73 | 0.75 | 1 | 7,842 |
|  | OF_S_B_1_I, III | *Percopsis omiscomaycus* | - | - | X | - | - | 1 | 1,536 |
|  | OF_S_B_1_II | *Notropis hudsonius* | X | - | X | 0.93 | - | 1 | 9,162 |
|  | OF_S_B_1_I, II, III | *Coregonus spp.* | X | X | - | 0.91 | 0.95 | - | 4,711 |
|  |  |  |  |  |  |  |  |  |  |
| OldFort | OF_1_I, II | *Lota lota* | X | - | - | 1 | - | - | 5 |
| (N=8) | OF_1_I, II, III | *Esox lucius* | X | X | X | 1 | 0.99 | 1 | 6,915 |
|  | OF_1_I, II, III | *Notropis atherinoides* | X | - | - | 1 | - | - | 20 |
|  | OF_1_I, II, III | *Sander vitreus* | X | X | - | 1 | 1 | - | 8,998 |
|  | OF_1_II | *Hiodon alosoides* | X | - | - | 1 | - | - | 10,803 |
|  | OF_1_I, III | *Catostomus commersonii* | - | - | X | - | - | 1 | 13,611 |
|  | OF_1_I, II, III | *Percopsis omiscomaycus* | - | - | X | - | - | 1 | 337 |
|  | OF_1 | *Coregonus spp.* | - | X | - | - | 0.95 | - | 12,394 |
|  |  |  |  |  |  |  |  |  |  |
| PeaceRiverA | PR_A_1_I, II, III | *Esox lucius* | X | X | X | 0.99 | 0.99 | 1 | 19,990 |
| (N=10) | PR_A_1_I, II, III | *Notropis atherinoides* | X | X | - | 1 | 0.98 | - | 12,403 |
|  | PR_A_1_I | *Sander vitreus* | X | - | X | 1 | - | 1 | 6,016 |
|  | PR_A_1_II, III | *Hiodon alosoides* | X | - | X | 1 | - | 0.996 | 3,153 |
|  | PR_A_1_III | *Lota lota* | X | - | - | 1 | - | - | 7,012 |
|  | PR_A_1_III | *Notropis hudsonius* | X | - | X | 0.93 | - | 1 | 8,541 |
|  | PR_A_1_III | *Percopsis omiscomaycus* | - | - | X | - | - | 1 | 119 |
|  | PR_A_1_III | *Catostomus commersonii* | X | - | X | 0.73 | - | 1 | 4,556 |
|  | PR_A_1_I | *Catostomus catostomus* | X | X | - | 0.95 | 0.99 | - | 7,604 |
|  | PR_A_1_I | *Coregonus spp.* | - | - | X | - | - | 0 | 67 |
|  |  |  |  |  |  |  |  |  |  |
| PeaceRiverB | PR_B_1_I, II, III | *Esox lucius* | X | - | X | 0.99 | - | 1 | 120 |
| (N=7) | PR_B_1_I, II, III | *Notropis atherinoides* | X | X | X | 1 | 0.98 | 1 | 13,850 |
|  | PR_B_1_I, II, III | *Perca flavescens* | X | - | - | 1 | - | - | 7 |
|  | PR_B_1_I, II, III | *Hiodon alosoides* | X | X | X | 1 | 1 | 1 | 2,596 |
|  | PR_B_1_II | *Catostomus catostomus* | X | - | - | 0.99 | - | - | 3 |
|  | PR_B_1_II, III | *Lota lota* | X | - | - | 1 | - | - | 2,926 |
|  | PR_B_1_II | *Sander vitreus* | X | - | - | 1 | - | - | 6 |
|  |  |  |  |  |  |  |  |  |  |
| PelicanCreek | P_CR_1_I, III | *Lota lota* | X | X | - | 1 | 1 | - | 808 |
| (N=9) | P_CR_1_I, II, III | *Esox lucius* | X | X | X | 0.99 | 0.99 | 1 | 19,831 |
|  | P_CR_1_I, II, III | *Notropis atherinoides* | X | X | - | 1 | 0.98 | - | 27,079 |
|  | P_CR_1_I, II, III | *Sander vitreus* | X | - | - | 1 | - | - | 6 |
|  | P_CR_1_I, II, III | *Hiodon alosoides* | X | X | X | 1 | 1 | 1 | 5,594 |
|  | P_CR_1_I, II, III | *Percopsis omiscomaycus* | - | - | X | - | - | 1 | 4,089 |
|  | P_CR_1_I,II | *Catostomus commersonii* | X | - | X | 0.73 | - | 1 | 2,428 |
|  | P_CR_1_I, II, III | *Rhinichthys cataractae* | X | - | - | 0.91 | - | - | 3 |
|  | P_CR_1_II, III | *Coregonus spp.* | X | X | - | 0.92 | 0.95 | - | 1,203 |
|  |  |  |  |  |  |  |  |  |  |
| PrairieRiverPC | PC_1_I, II, III | *Lota lota* | X | - | - | 1 | - | - | 12 |
| (N=9) | PC_1_I, II, III | *Esox lucius* | X | X | X | 0.99 | 0.99 | 1 | 117,500 |
|  | PC_1_I, II, III | *Notropis atherinoides* | X | X | - | 1 | 0.98 | - | 79,572 |
|  | PC_1_I, II, III | *Sander vitreus* | X | - | X | 1 | - | 1 | 1,434 |
|  | PC_1_I, II, III | *Hiodon alosoides* | X | X | X | 1 | 1 | 0.994 | 9,323 |
|  | PC_1_I, II, III | *Notropis hudsonius* | X | X | - | 0.93 | 0.99 | - | 603 |
|  | PC_1_I, II, III | *Percopsis omiscomaycus* | - | - | X | - | - | 1 | 6,562 |
|  | PC_1_I, III | *Catostomus commersonii* | X | - | X | 0.73 | - | 1 | 7 |
|  | PC_1_II | *Rhinichthys cataractae* | X | - | - | 0.91 | - | - | 3 |
|  |  |  |  |  |  |  |  |  |  |
| QuatreFourches | QS_1_I, II, III | *Lota lota* | X | X | - | 1 | 1 | - | 3,416 |
| (N=10) | QS_1_I, II, III | *Esox lucius* | X | X | X | 0.99 | 0.99 | 1 | 57,329 |
|  | QS_1_I, II, III | *Notropis atherinoides* | X | X | - | 1 | 0.98 | - | 47,815 |
|  | QS_1_I, II | *Perca flavescens* | X | - | - | 1 | - | - | 3 |
|  | QS_1_I, II, III | *Hiodon alosoides* | X | X | X | 1 | 1 | 1 | 5,173 |
|  | QS_1_II | *Sander vitreus* | X | X | - | 1 | 1 | - | 880 |
|  | QS_1_II, III | *Notropis hudsonius* | X | - | - | 0.99 | - | - | 7,186 |
|  | QS_1_I, II, III | *Percopsis omiscomaycus* | - | - | X | - | - | 1 | 249 |
|  | QS_1_I | *Catostomus commersonii* | X | X | X | 0.73 | 0.75 | 1 | 5 |
|  | QS_3 | *Coregonus spp.* | - | X | - | - | 0.95 | - | 3,334 |
|  |  |  |  |  |  |  |  |  |  |
| RapidsA | R_A_1_I, II, III | *Catostomus catostomus* | X | - | - | 0.99 | - | - | 1,858 |
| (N=11) | R_A_1_I, II, III | *Lota lota* | X | X | - | 1 | 1 | - | 4,784 |
|  | R_A_1_I, II, III | *Esox lucius* | X | X | X | 0.99 | 0.99 | 1 | 105,898 |
|  | R_A_1_I, II, III | *Notropis atherinoides* | X | X | X | 1 | 0.98 | 1 | 39,639 |
|  | R_A_1_I, II, III | *Sander vitreus* | X | - | - | 1 | - | - | 2,421 |
|  | R_A_1_I, II | *Perca flavescens* | X | X | - | 1 | 1 | - | 7,819 |
|  | R_A_1_I, II, III | *Notropis hudsonius* | X | X | X | 0.99 | 0.93 | 1 | 5 |
|  | R_A_1_I, II, III | *Hiodon alosoides* | X | - | X | 1 | - | 1 | 8,477 |
|  | R_A_1_I, II | *Percopsis omiscomaycus* | - | - | X | - | - | 1 | 173 |
|  | R_A_1_I, III | *Catostomus commersonii* | X | X | X | 0.73 | 0.75 | 1 | 6,667 |
|  | R_A_1_III | *Coregonus spp.* | X | - | - | 0.91 | - | - | 6,562 |
|  |  |  |  |  |  |  |  |  |  |
| Big Egg Lake | BEL_1_I, II | *Esox lucius* | X | X | - | 0.99 | 0.99 | - | 1,715 |
| (N=4) | BEL_1_I, II, III | *Hiodon alosoides* | - | X | - | - | 1 | - | 1,200 |
|  | BEL_1_II, III | *Oncorhynchus spp.* | X | - | - | 0 | - | - | 18 |
|  | BEL_1_II, III | *Coregonus spp.* | X | X | - | 0.92 | 0.95 | - | 2,452 |
